# Contrasting evolutionary histories of the legless lizards slow worms (*Anguis*) shaped by the topography of the Balkan Peninsula

**DOI:** 10.1186/s12862-016-0669-1

**Published:** 2016-05-10

**Authors:** Daniel Jablonski, David Jandzik, Peter Mikulíček, Georg Džukić, Katarina Ljubisavljević, Nikolay Tzankov, Dušan Jelić, Evanthia Thanou, Jiří Moravec, Václav Gvoždík

**Affiliations:** Department of Zoology, Comenius University in Bratislava, Mlynská dolina, Ilkovičova 6, 842 15 Bratislava, Slovakia; Department of Ecology and Evolutionary Biology (EBIO), University of Colorado, Ramaley N122, Campus, Box 334, 80309-0334 Boulder, CO USA; Department of Evolutionary Biology, Institute for Biological Research “Siniša Stanković”, 11060 Belgrade, Serbia; Department of Vertebrates, National Museum of Natural History, Tsar Osvoboditel Blvd. 1, 1000 Sofia, Bulgaria; Croatian Institute for Biodiversity, Croatian Herpetological Society Hyla, I, Breznička 5a, 10000 Zagreb, Croatia; Department of Biology, Section of Animal Biology, School of Natural Sciences, University of Patras, GR-26500 Patras, Greece; Department of Zoology, National Museum, 193 00 Prague, Czech Republic; Institute of Vertebrate Biology, Czech Academy of Sciences, 603 65 Brno, Czech Republic

**Keywords:** Anguidae, Squamata, Phylogeography, Biogeography, Speciation, Contact zones, Microrefugia, Balkan mountains

## Abstract

**Background:**

Genetic architecture of a species is a result of historical changes in population size and extent of distribution related to climatic and environmental factors and contemporary processes of dispersal and gene flow. Population-size and range contractions, expansions and shifts have a substantial effect on genetic diversity and intraspecific divergence, which is further shaped by gene-flow limiting barriers. The Balkans, as one of the most important sources of European biodiversity, is a region where many temperate species persisted during the Pleistocene glaciations and where high topographic heterogeneity offers suitable conditions for local adaptations of populations. In this study, we investigated the phylogeographical patterns and demographic histories of four species of semifossorial slow-worm lizards (genus *Anguis*) present in the Balkan Peninsula, and tested the relationship between genetic diversity and topographic heterogeneity of the inhabited ranges.

**Results:**

We inferred phylogenetic relationships, compared genetic structure and historical demography of slow worms using nucleotide sequence variation of mitochondrial DNA. Four *Anguis* species with mostly parapatric distributions occur in the Balkan Peninsula. They show different levels of genetic diversity. A signature of population growth was detected in all four species but with various courses in particular populations. We found a strong correlation between genetic diversity of slow-worm populations and topographic ruggedness of the ranges (mountain systems) they inhabit. Areas with more rugged terrain harbour higher genetic diversity.

**Conclusions:**

Phylogeographical pattern of the genus *Anguis* in the Balkans is concordant with the refugia-within-refugia model previously proposed for both several other taxa in the region and other main European Peninsulas. While slow-worm populations from the southern refugia mostly have restricted distributions and have not dispersed much from their refugial areas, populations from the extra-Mediterranean refugia in northern parts of the Balkans have colonized vast areas of eastern, central, and western Europe. Besides climatic historical events, the heterogeneous topography of the Balkans has also played an important role in shaping genetic diversity of slow worms.

**Electronic supplementary material:**

The online version of this article (doi:10.1186/s12862-016-0669-1) contains supplementary material, which is available to authorized users.

## Background

Diversity of European biota has been strongly influenced by global climatic and environmental changes in the Quaternary. Toward the end of the Pleistocene, repeated climatic oscillations led to extinctions of many phylogenetic lineages from vast northern areas during glacial periods followed by re-colonisations during interglacials [1–3]. Many plant and animal lineages survived cold and dry glacials in relatively stable and hospitable environments. In Europe these were located in three Mediterranean peninsulas: Iberian, Italian, and Balkan. This general biogeographical model has been expanded to a more complex view acknowledging long-term persistence of cold-tolerant species in central and northern Europe during glacials and survival in multiple refugia located within the Mediterranean peninsulas [4, 5]. Demographic stability of populations in southern refugia enabled them to diverge, which has resulted in high diversity in all three main refugial regions. In contrast, northern populations established during re-colonization are generally characterized by lower taxonomic and genetic diversity.

In comparison to the Iberian and Italian peninsulas, the Balkans has remained much less studied in terms of the biogeographical history of the species distributed there, although it is richer both in biodiversity and paleoendemics [6–8]. The Balkan Peninsula is not isolated by one extended mountain range such as the Pyrenees of the Iberian and the Alps of the Italian Peninsula, and so there are fewer dispersal barriers to the north. This allowed postglacial expansion of populations from the Balkan refugia to central and northern Europe [1, 5, 9]. On the other hand, the Balkan Peninsula is a region with high topographic and climatic heterogeneity, showing a strong contrast between the eastern/western and northern/southern parts. In the east and north, the surface is formed by plains or plateaus and the mountain slopes are generally gentle, while in the west and south the Dinarides and Hellenides rise steeply from the coastal strip [10]. Each of the Balkan mountain chains also has a different tectonic and sedimentary history, and while they all underwent complex folding and faulting in the process of the Alpine orogenesis, the intensity was different [11]. All this geographical variation offers suitable conditions for local adaptations of populations, which could promote divergence and subsequent diversification [12, 13].

We, and others [14–17] have been studying the evolutionary history of legless lizards of the genus *Anguis* (family Anguidae) within its Western Palearctic range. This genus comprises five species, four of which occur in the Balkans [14, 15]. While *Anguis cephallonica* Werner, 1894 and *A. graeca* Bedriaga, 1881 are Balkan endemics with rather restricted distribution in the south of the peninsula, ranges of *A. fragilis* Linnaeus, 1758 and *A. colchica* (Nordmann, 1840) are at the continental scale and cover vast areas of Europe and western Asia [14, 17–19]. Considering the semifossorial lifestyle and high site tenacity [20, 21], one might expect restricted occurrence of slow worms. However, distribution of slow worms in the Balkan Peninsula seems to be more or less continuous with gaps probably only in agricultural regions and extremely high altitudes [19, 22, 23]. Nevertheless, details of the species ranges within the Balkans, contact zones of multiple species, and detailed intraspecific genetic structure in respect to geography and ecology still remain widely unknown.

In this study we collected and analysed data originating from the Balkan slow-worm populations with the aim to i) provide a detailed picture of distribution; ii) infer historical relationships of populations and describe genetic diversity; iii) reconstruct biogeographical histories of the Balkan slow-worm populations during the Quaternary. Finally, we tested iv) whether the genetic diversity observed in the Balkan slow worms is driven by specifics of topography. Dispersal barriers would most likely coincide with the extensive and variously rugged mountain ranges of the Balkan Peninsula, thus we expected the slow-worm genetic diversity to be correlated with topographic variation of this region.

## Methods

### Sampling

**Fig. 1:**
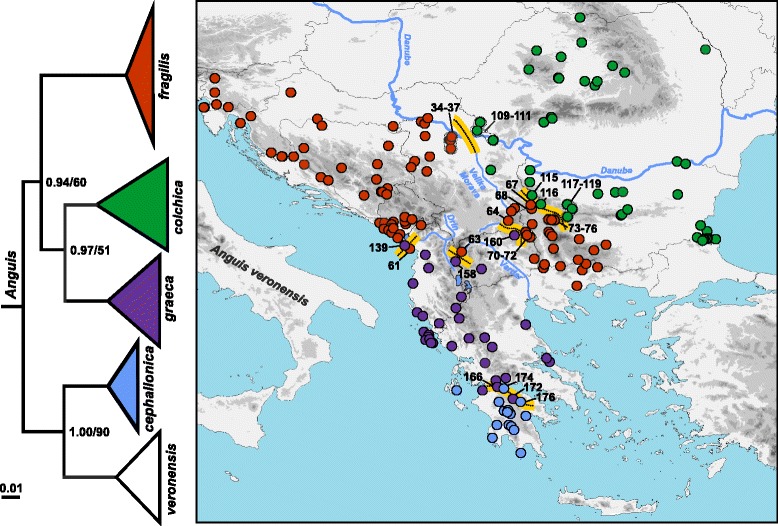
Maximum likelihood (ML) phylogenetic tree of *Anguis* species and their distributions in the Balkans based on a fragment of mtDNA (*ND2*). *Anguis veronensis* (in white) occurs outside the area studied here. Numbers at nodes show Bayesian posterior probabilities and ML bootstrap support values. Yellow lines denote contact zones between two species. Numbers correspond to the locality numbers as given in Additional file 1: Table S1

Since the Balkan Peninsula represents an important evolutionary centre of the genus *Anguis*, we devoted this study to slow-worm populations from this region. Our sampling strategy focused on equally representing the whole Balkan region as well as all four Balkan species. Sampling effort also took into account that these species vary in distribution ranges and inter-specific genetic diversification and may have low population densities in some areas. Tissue samples were obtained mainly from road-killed individuals or alternatively from living animals as oral swabs, blood droplets, or miniature skin biopsies. This sampling procedure did not affect survival of the captured animals. No experimental research was carried out on these animals in this study. All samples were preserved in 96 % ethanol. A portion, 732 base pairs (bp), of the mitochondrial gene for NADH dehydrogenase subunit 2 (*ND2*) was targeted. Newly produced nucleotide sequences were supplemented to previously published sequences from the Balkans [14–17] to complete a total of 231 specimens from 187 localities. Based on the mtDNA identity, we represented all four Balkan *Anguis* species, namely 110 *A. fragilis*, 56 *A. colchica*, 49 *A. graeca*, 16 *A. cephallonica* (Fig. 1; Additional file 1: Table S1). To put our Balkan data into a complex phylogeographical context, we compiled an additional dataset supplemented by all known haplotypes, including those from outside the Balkans, previously published by [14, 15]: *A. fragilis* (f1–f15), *A. colchica* (c1–c12), *A. graeca* (g1–g16), *A. cephallonica* (ce1, ce2), *A. veronensis* (v1–15); and [16]: *A. fragilis* (AF01–AF07), *A. colchica* (AC01, AC02). The resulting dataset contained 271 sequences, excluding outgroup. Following previous works of our team [14, 15], we used the sister genus *Pseudopus* as outgroup (*P. apodus thracius* from Albania, Pat1, GenBank No. FJ666589).

### Laboratory procedures

Total genomic DNA was extracted using various commercial kits and following respective manufacturer protocols. We amplified > 1400 bp-long portion of mtDNA comprising the complete *ND2* gene, five subsequent transfer RNA (*tRNAs*) genes and the light-strand replication origin using primers (L4437n, H5934) and protocol following [14]. We sequenced only the first half of the amplicon using the internal reverse primer AND2inR2 [14], which was also used in PCR amplifications in cases of samples with degraded DNA, using the same protocol. Alternatively, the internal reverse primer AND2inRc [14] was used in *A. cephallonica* for both PCR amplifications (in degraded DNA) and sequencing. The final stretch contained 732 bp-long fragment of *ND2* after trimming the low quality ends. The sequencing was performed by Macrogen Inc. (Seoul, South Korea or Amsterdam, Netherlands; http://www.macrogen.com) and new sequences have been deposited in GenBank under accession numbers KX020147–KX020322 (Additional file 1: Table S1).

### DNA sequence evaluation, phylogenetic analyses, and haplotype networks

The protein-coding *ND2* fragments (732 bp) were aligned manually. No stop codons were detected when the sequences were translated using the vertebrate mitochondrial genetic code in the program DnaSP 5.10 [24]. The same program was used to calculate uncorrected *p*-distances among the main lineages or haplogroups within each taxon, and to estimate the number of haplotypes (*h*), haplotype diversity (*Hd*), number of segregating sites (*S*), nucleotide diversity (*π*), and Watterson’s theta (*θ*_W_) for each of these lineages or haplogroups.

For phylogenetic analyses we used the all-individuals dataset supplemented by distinct published haplotypes from outside the Balkans to obtain a complex picture of the phylogenetic relationships within the Balkan Peninsula and in the framework of the whole genus. The best-fit codon-partitioning schemes and the best-fit substitution models were selected using PartitionFinder v1.1.1. [25], according to the Bayesian information criterion (BIC), separately for each dataset and methodological approach (i.e. models available in the used software). Phylogenetic trees were inferred using the Bayesian approach (BA) and maximum likelihood (ML) with the software MrBayes 3.2 [26] and RAxML 8.0 [27], respectively. Each codon position treated separately was selected as the best-fit partitioning scheme for both BA and ML with the best-fit substitution models for the BA analysis as follows: HKY + G (1^st^ codon position), HKY + I (2^nd^ codon position), and HKY + G (3^rd^ codon position); and for the ML analysis: GTR + G in each codon position. The ML clade support was assessed by 1,000 bootstrap pseudoreplicates. The MrBayes analysis was set as follows: two separate runs, with four chains for each run, 10 million generations with samples saved every 100^th^ generation. The convergence of the two runs was confirmed by the convergence diagnostics (average standard deviation of split frequencies, potential scale reduction factor). First 20 % of trees were discarded as the burn-in after inspection for stationarity of log-likelihood scores of sampled trees in Tracer 1.6 [28] (all parameters had effective sample size > 200). Majority-rule consensus tree was drawn from the post-burn-in samples and posterior probabilities were calculated as the frequency of samples recovering any particular clade.

Haplotype-network approaches can be more effective for presentation of intraspecific evolution than the tree-based phylogenetic approaches [29]. Therefore, we also constructed haplotype networks for individual species (or main clades in *A. colchica*) using the 95 % limit of parsimony as implemented in TCS 1.21 [30]. To infer possible connections to a network when cases of highly divergent haplotypes were detected (two haplotypes in *A. graeca*, and one in *A. cephallonica*), we also applied a fixed connection limit at a higher number of steps allowing visualization of their likely connections to the networks constructed under the 95 % limit of parsimony.

### Demographic analyses

The past population dynamics of the main population groups were inferred using the Bayesian coalescent-based approach of the Bayesian skyline plots (BSPs; [31]) as implemented in BEAST 2.1 [32]. This method computes the effective population size (*N*_*e*_) through time directly from sampled sequences and does not require a specific *a priori* assumed demographic model. Main population groups correspond to monophyletic groups. In a single case of several basal haplogroups of *A. fragilis*, the population group was defined geographically (‘Slovenian’ populations). Preliminary analyses were run using both strict molecular clock and uncorrelated lognormal relaxed molecular clock. Since the parameter of the standard deviation of the uncorrelated lognormal relaxed clock was close to zero, the final analyses were run enforcing the strict molecular clock model. A uniform prior for the substitution rate with the initial value 0.0065 substitution/site/lineage/Myr (as suggested for the used mtDNA marker in anguid lizards; [33]) was set as no internal calibration point was available. Using PartitionFinder v1.1.1. [25], all codon positions treated together as one partition and the HKY substitution model were selected as the best-fit partitioning scheme and the best-fit model, respectively, for each population group. The final BSP analyses were run in duplicates to check for consistency between runs, each for at least 10 million generations (or more according to each dataset until the effective sample size [ESS] > 200 was achieved) and sampled every 1000 generations (or more, accordingly, to save 10,000 samples). Convergence, ESS, stationarity, and the appropriate number of generations to be discarded as burn-in (10 %) were assessed using Tracer 1.6 [28]. The resulting BSPs were also summarized in Tracer 1.6 with the maximum times as the median of the root height parameter.

In addition, the mismatch distributions (MD) were calculated as the distributions of the observed pairwise nucleotide differences and the expected values under a growing- or declining-population model using DnaSP 5.10 [24]. The occurrence of historical demographic changes was assessed by the neutrality-test statistics of the Fu’s *F*_*S*_ [34] Tajima’s *D* [35], and Ramos-Onsins and Rozas’s *R*_2_ [36] calculated in DnaSP 5.10 with the estimation of the statistical significance using 10,000 coalescent simulations.

### Genetic diversity and topographic heterogeneity

Since a more complex topography is more likely to limit dispersal and gene flow, we hypothesized that regions with higher topographic heterogeneity (terrain ruggedness) will be inhabited by slow-worm lineages characterized by higher genetic diversity. To test for this relationship we performed regression analyses of nucleotide diversity (*π*) with the terrain ruggedness index (TRI). TRI is a measure of topographic heterogeneity calculated as a sum change in elevation between a grid cell and its eight neighbour cells in a grid network [37]. Cell TRI values are then averaged across specific areas such as mountains. Values of TRI were derived from digital elevation model based on the data from the NASA Shuttle Radar Topographic Mission (SRTM-3; available at http://srtm.usgs.gov) with a spatial resolution of approximately 3 arc-sec (~100 × 100 m) with a final resample to 30 arc-sec (~1 × 1 km) using GRASS GIS 7.1 [38]. The polygon network was created for the selected topographic units, mountain ranges, which respect distributions of evolutionary lineages or haplogroups (Apuseni Mts., Carpathians, Dinarides, Hellenides, Prealps, Peloponnese, Macedonian-Thracian Massif, Stara Planina Mts.; Additional file 2: Table S2, Additional file 3: Figure S1 and Additional file 4: Table S3). Since higher genetic diversity might be expected in geographically larger areas, we controlled for the effect of the topographic-unit size. In the multiple linear regressions, we regressed nucleotide diversity of individual phylogenetic lineages/haplogroups against ‘extreme’ values of TRI calculated for the topographic units (‘extreme’ values were taken from the highest 25 % of data: 3rd quartile (Q3), and median and modus of the values above Q3), and against topographic-unit sizes (in km^2^). The ‘extreme’ values of TRI were selected with the aim to preferentially study the influence of steeper terrain, presumably posing stronger barriers to gene flow and resulting thus in higher probability of lineage divergence. Due to the controversy about the biogeographical significance of the Apuseni Mts. as a separate unit within the Carpathians [39, 40], we performed two separate analyses with samples from the Apuseni Mts. included and excluded, respectively, within the group of the Carpathian samples. The GIS analyses were performed using ArcGIS 10.1 (ESRI) and the multiple linear regressions were carried out using STATISTICA version 12 [41].

## Results

### Phylogeny, species distributions and contact zones

The maximum likelihood and Bayesian phylogenetic analysis provided topologies concordant with previous studies [14, 15, 17]. The southernmost species, *A. cephallonica*, forms a clade with *A. veronensis* from the Italian Peninsula, while the other three species (*A. fragilis*, *A. colchica*, and *A. graeca*) form a separate clade, in which the Balkan endemic *A. graeca* is in a sister position to the eastern widespread species, *A. colchica* (Fig. 1).

*Anguis fragilis* is distributed in the northwestern and central parts of the Balkan Peninsula from the Julian Alps and the southeastern Prealps, along the Dinarides to the Macedonian-Thracian Massif, and only marginally in the northern Hellenides (Figs. 1 and 10). *Anguis colchica* is documented from the Carpathians, the Balkanides along the Stara Planina Mts. in southeastern Serbia and central Bulgaria, and from the Black Sea region (Strandzha Mts.). *Anguis graeca* is mostly confined to the Hellenides in the southern Balkans where it is distributed from the northern Peloponnese, along the Pindus Mts. and the Albanian Mts. to the southernmost Dinaric region (southern Montenegro) and western Macedonian-Thracian Massif (northeastern Rep. Macedonia). *Anguis cephallonica* was found in the Peloponnese and Kephallonia Island (not sampled in Zakynthos and Ithaki islands in this study where the species was documented previously [42, 43]).

Our detailed sampling also revealed several areas where haplotypes of different species could be found in distances from ca. 15 to 80 km, indicating the existence of contact zones (Fig. 1). One such contact zone between *A. fragilis* and *A. colchica* was detected in eastern and southeastern Serbia and central-western Bulgaria (sites 34–37, 67–68, 73–76 for *A. fragilis*; sites 109–111, 115–116, 117–119 for *A. colchica*). Three zones of contact were further detected between *A. fragilis* and *A. graeca* in southernmost Montenegro (sites 61 and 139; sympatric occurrence), northwestern Rep. Macedonia (sites 63 and 158), and in the tri-border area of Serbia, Bulgaria and Rep. Macedonia (sites 64, 70–72, 160). Sympatric occurrence of *A. graeca* and *A. cephallonica* was confirmed from northern Peloponnese (sites 165, 166, 172; and 174–176).

### Genetic diversity and phylogeographical patterns

The dataset built up from the Balkan specimens contained 231 ingroup (*Anguis*) sequences, which yielded a total of 100 haplotypes. Nucleotide diversity was higher in the two Balkan endemics, *A. graeca* (*π* = 1.17 ± 0.11 %) and *A. cephallonica* (*π* = 0.81 ± 0.21 %), than in the Balkan populations of the two northerly distributed taxa, *A. colchica* (*Incerta* clade; *π* = 0.66 ± 0.05 %) and *A. fragilis* (*π* = 0.34 ± 0.04 %; Table 1).

**Table 1:** Summary of genetic polymorphism and results of neutrality tests for the Balkan populations of four species of the genus *Anguis*

Species/clade/lineage/haplogroup	*n*	*h*	*S*	*π* ± SD (%)	*h*_*d*_ ± SD	*θ*_*W*_ ± SD (%)	*F*_*S*_	*P* [*F*_*S*_]	*R*_*2*_	*P* [*R*_*2*_]	*D*	*P* [*D*]
***A. cephallonica***	16	13	33	0.81 ± 0.21	0.980 ± 0.030	1.36 ± 0.53	–	–	–	–	–	–
Mani lineage	1	1	–	–	–	–	–		–	–	–	–
Widespread lineage	15	12	19	0.59 ± 0.08	0.971 ± 0.033	0.8 0 ± 0.33	**–5.578**	0.003	**0.0824**	0.005	–1.087	0.139
***A. colchica***	56	24	57	2.00 ± 0.22	0.894 ± 0.033	1.70 ± 0.50						–
PONTIC clade	13	8	10	0.29 ± 0.07	0.885 ± 0.070	0.44 ± 0.21	**–3.410**	0.011	**0.0993**	0.013	–1.357	0.083
*INCERTA* clade	43	16	27	0.66 ± 0.05	0.829 ± 0.052	0.85 ± 0.29	–2.632	0.175	0.0837	0.218	–0.753	0.256
Stara-Planina lineage	22	5	5	0.09 ± 0.03	0.407 ± 0.128	0.19 ± 0.10	**–2.263**	0.017	**0.0769**	0.001	**–1.631**	0.017
Banatian lineage	3	3	4	0.36 ± 0.11	1.000 ± 0.272	0.36 ± 0.26	–	–	–	–	–	–
Carpathian lineage	18	8	12	0.47 ± 0.04	0.889 ± 0.042	0.48 ± 0.21	–0.736	0.356	0.1320	0.0441	–0.082	0.510
***A. fragilis***	110	34	49	0.34 ± 0.04	0.851 ± 0.028	1.28 ± 0.35	–	–	–	–	–	–
Carniolan	2	2	2	0.27 ± 0.14	1.000 ± 0.500	0.27 ± 0.24	–	–	–	–	–	–
Alpine-Pannonian	2	1	–	–	–	–	–	–	–	–	–	–
North Adriatic	3	2	1	0.09 ± 0.04	0.667 ± 0.314	0.09 ± 0.09	–	–	–	–	–	–
‘Slovenian’ haplogroups together	7	5	11	0.68 ± 0.10	0.905 ± 0.103	0.62 ± 0.32	0.276	0.507	0.1976	0.506	0.557	0.722
Illyrian-Central European	71	22	29	0.21 ± 0.03	0.706 ± 0.059	0.82 ± 0.26	–	–	–	–	**–**	–
South Balkan	32	7	6	0.13 ± 0.02	0.679 ± 0.065	0.20 ± 0.10	**–2.853**	0.025	0.0797	0.089	–1.034	0.174
ICE ± SB	103	29	36	0.25 ± 0.02	0.831 ± 0.031	0.95 ± 0.27	**–26.331**	< 0.001	**0.0253**	0.001	**–2.257**	0.001
***A. graeca***	49	29	73	1.17 ± 0.11	0.964 ± 0.013	2.24 ± 0.66	**–9.390**	0.010	**0.0522**	0.013	**–1.703**	0.023
*graeca* XII	1	1	–	–	–	–	–	–	–	–	–	–
*graeca* XI	5	1	–	–	–	–	–	–	–	–	–	–
*graeca* X	1	1		–	–	–	–	–	–	–	–	–
*graeca* IX	1	1	–	–	–	–	–	–	–	–	–	–
*graeca* VIII	1	1	–	–	–	–	–	–	–	–	–	–
*graeca* VII	1	1	–	–	–	–	–	–	–	–	–	–
*graeca* VI	1	1	–	–	–	–	–	–	–	–	–	–
*graeca* V	14	8	11	0.37 ± 0.08	0.901 ± 0.058	0.47 ± 0.22	–	–	–	–	–	–
*graeca* IV	4	3	5	0.36 ± 0.12	0.833 ± 0.222	0.37 ± 0.24	–	–	–	–	–	–
*graeca* III	1	1	–	–	–	–	–	–	–	–	–	–
*graeca* II	2	2	3	0.41 ± 0.21	1.000 ± 0.500	0.41 ± 0.34	–	–	–	–	–	–
*graeca* I	15	6	9	0.27 ± 0.05	0.790 ± 0.079	0.38 ± 0.18	–	–	–	–	–	–
KJ634800	1	1	–	–	–	–	–	–	–	–	–	–
KJ634801	1	1	–	–	–	–	–	–	–	–	–	–

Underlined populations were included in the demographic analyses. Sample size (*n*), number of haplotypes (*h*), number of polymorphic sites (*S*), nucleotide diversity (*π*), haplotype diversity (*h*_*d*_), Watterson’s theta per site (*θ*_*W*_), Fu’s *F*_*S*_ statistics (*F*_*S*_), Ramos-Onsins and Rozas’s *R*_2_ statistics (*R*_2_), Tajima’s *D* statistics (*D*), and their probability values (*P*) are given. Values marked in bold are statistically significant. SD = standard deviation

**Fig. 2:**
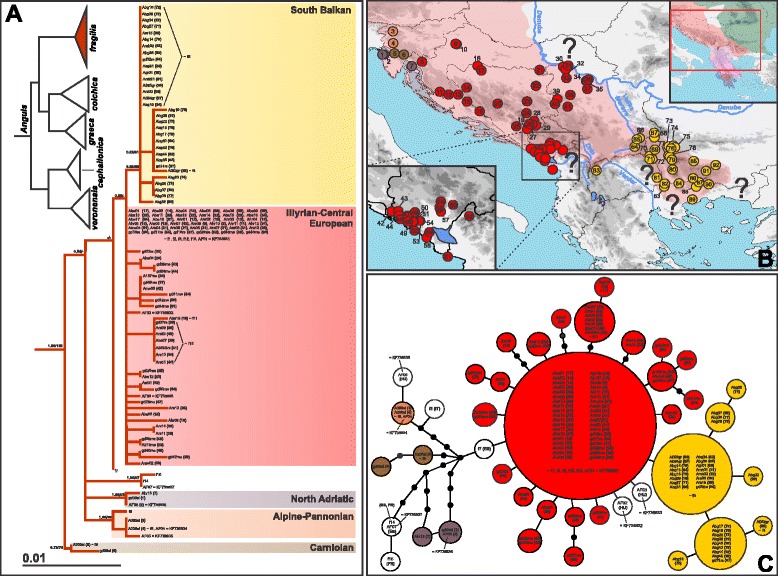
*Anguis fragilis*, (**a**) maximum likelihood (ML) phylogeny, (**b**) geographical distributions, and (**c**) parsimony haplotype network of the main haplogroups in the Balkans. Numbers at nodes in the tree represent Bayesian posterior probabilities (pp) and ML bootstrap support values (pp below 0.50 and bootstrap values below 50 are not shown). Locality numbers (in parentheses) follow sample IDs and correspond to the numbers in Additional file 1: Table S1. White circles in the network represent extralimital haplotypes as detected in previous studies (Gvoždík et al. [14, 15]; Szabó & Vörös, [16]; Thanou et al. [17])

*Anguis fragilis* shows relatively low genetic variation, with 34 haplotypes identified among 110 individuals (intraspecific *p*-distance ≤ 1.1 %; Additional file 5: Table S4; Fig. 2). The basal radiation was detected in the northwest of the Balkans, in the northern Dinarides and southeastern Prealps (sites 1–7). Haplotypes from this basal radiation do not form a monophylum and may be divided into three Slovenian haplogroups, which we name in accordance to the detected distributions as follows: North Adriatic (sites 1, 2, 7); Carniolan (sites 5, 6); and Alpine-Pannonian (sites 3, 4). In earlier studies, haplotypes belonging to the latter haplogroup were also found outside the Balkans, i.e. in northeastern Italy (haplotype f8 – [15]) and the Pannonian Plain (haplotypes AF04, AF05 – [16]). Another haplogroup from the basal radiation (haplotypes f14, f15 – [15]; and AF07 – [16]) and a single haplotype (f7 [14]) conform to populations from Western Europe (Spain, France). All other *A. fragilis* haplotypes cluster into one large unit that might be divided into two geographically separated haplogroups: the northern we hereafter name the Illyrian-Central European haplogroup (ICE), and the southern one (the South Balkan haplogroup, SB). Haplotypes from the ICE haplogroup were also detected in Central Europe and southern Great Britain (haplotypes f1–f3, f12, f13 – [14, 15]; AF01–AF03 – [16]). The ICE haplogroup is paraphyletic in respect to the SB haplogroup. Nevertheless, the SB haplogroup is geographically well defined, confined to the Macedonian-Thracian Massif and only slightly penetrating to the northern Hellenides (Figs. 2b and 10). The ICE haplogroup is distributed along the Dinarides and surrounding lowland areas.

**Fig. 3:**
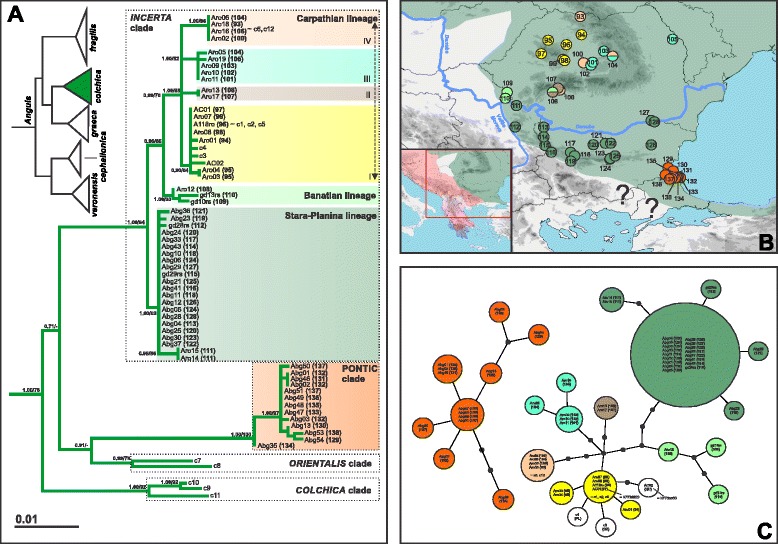
*Anguis colchica*, (**a**) maximum likelihood (ML) phylogeny, (**b**) geographical distribution of the main haplogroups in the Balkans, and (**c**) parsimony haplotype networks of the two Balkan clades (*Incerta* and Pontic). See the legend to Fig. 2 for more details

In *Anguis colchica*, a deep intraspecific divergence (4.3 % *p*-distance; Additional file 5: Table S4) was found separating two clades of a different geographical origin (Fig. 3a). One clade is widespread and corresponds to the subspecies *A. colchica incerta* [14], hereafter named the *Incerta* clade, while the second clade was detected in the Black Sea coastal area, therefore named the Pontic clade. Outside the Balkans, *A. colchica* forms two additional clades distributed in the Caucasus (*A. c. colchica*; the *Colchica* clade) and the southern Caspian region (*A. c. orientalis*; the *Orientalis* clade); see also [14]. The Pontic clade is currently only known from the Strandzha Mts. in southeastern Bulgaria. The mtDNA polymorphism of the Pontic clade is relatively high (8 haplotypes within 13 specimens) in respect to its restricted geographical range (Fig. 3b, c). The *Incerta* clade (16 haplotypes within 43 specimens) is widespread along the Carpathians and the Stara Planina Mts., with relatively high genetic variation and diversified into three main well-supported lineages: (i) Stara-Planina lineage in the region of the Stara Planina Mts. and the northern foothills, reaching the Serbian Carpathians (sites 111, 112); (ii) Banatian lineage detected in the Banat (southwestern Carpathians); and (iii) Carpathian lineage present in most of the Carpathians with a further sub-structure forming at least four haplogroups; Carpathian I–IV (Fig. 3a). The Carpathian I haplogroup seems to be confined to Transylvania (Apuseni Mts. and their vicinity; sites 94–99), while the other three are partially sympatric. The Carpathian lineage contains haplotypes that were also detected outside the Balkans in earlier studies (c1–c6, c12 – [14, 15]; AC01, AC02 – [16]; Fig. 3c).

**Fig. 4:**
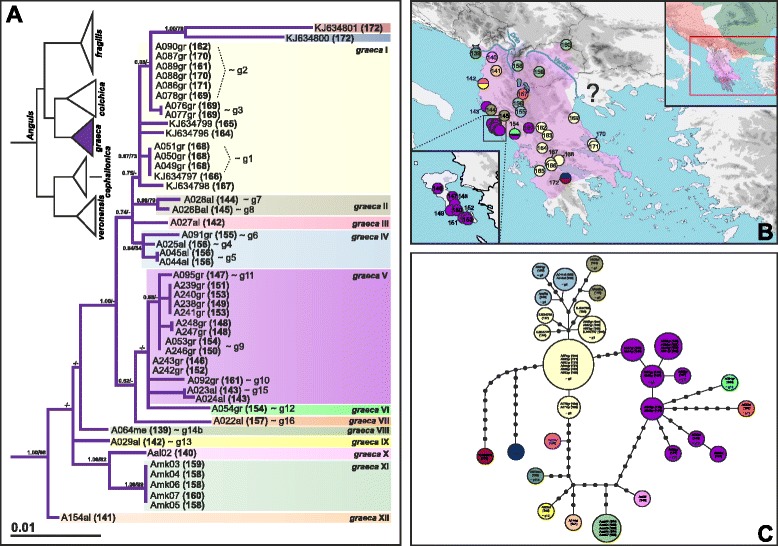
*Anguis graeca*, (**a**) maximum likelihood (ML) phylogeny, (**b**) geographical distribution, and (**c**) parsimony haplotype network of the main haplogroups. See the legend to Fig. 2 for more details

Of the two Balkan endemics, *A. graeca* shows a higher nucleotide but comparable haplotype diversity (29 haplotypes detected among 49 individuals) than the less widespread *A. cephallonica*. The genetic structure of *A. graeca* is complex, characterized by many haplogroups but without deep divergences (Fig. 4). Only two detected haplotypes (KJ634800, KJ634801) are relatively divergent both from each other and from all other haplotypes. They both originate from the same location in the northern Peloponnese (site 172). Geographical distributions of most haplogroups are restricted to small areas, mainly in the mountains of Albania (Fig. 4b). Only three haplogroups have wider distribution: one in central and southern mainland Greece, northern Peloponnese and Euboea Island (*graeca* I); the second in western Greece, Corfu Island and southern Albania (*graeca* V); and the third one in Rep. Macedonia (*graeca* XI).

**Fig. 5:**
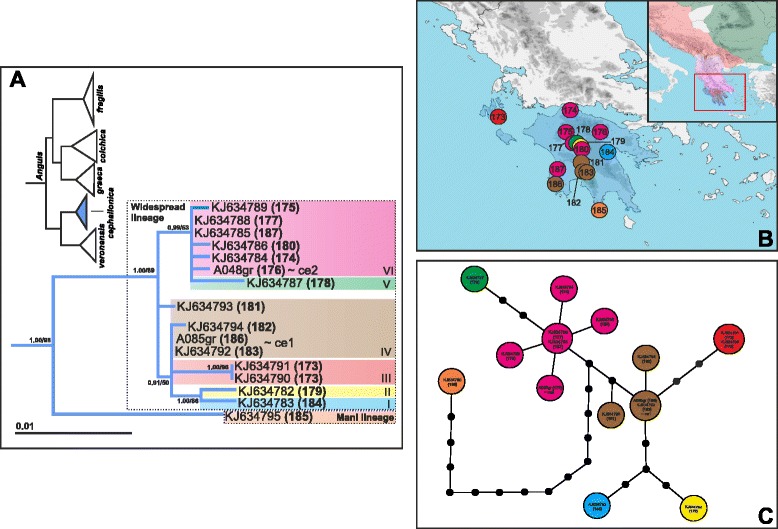
*Anguis cephallonica*, (**a**) ML phylogeny, (**b**) geographical distributions, and (**c**) parsimony haplotype network of the main haplogroups. See the legend to Fig. 2 for more details

The Peloponnese endemic, *A. cephallonica*, has a similarly complex phylogeographical structure with 13 haplotypes detected among 16 specimens (Fig. 5). One haplotype (KJ634795) originating from the Mani Peninsula in the south forms a lineage (hereafter the Mani lineage) divergent from all the other haplotypes, which form a well-supported monophylum (hereafter the Widespread lineage; 2.4 % *p*-distance; Additional file 5: Table S4; Fig. 5a). The Widespread lineage displays an inner diversification with several haplogroups distributed around the Peloponnese and Kephallonia Island with east–west longitudinal structure and higher diversity in the central Peloponnese (Fig. 5b, c).

### Historical demography

**Fig. 6:**
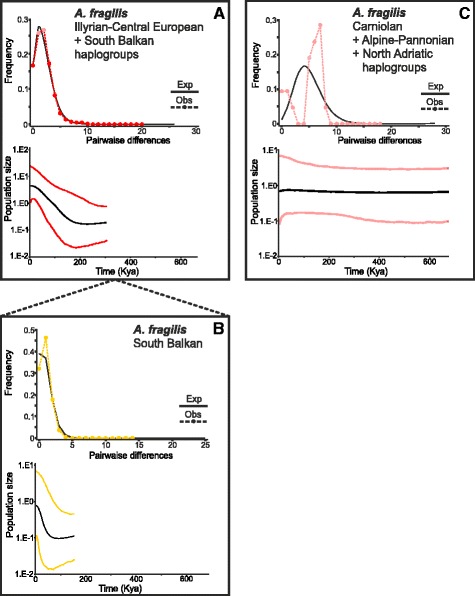
Mismatch distributions (MD) and Bayesian skyline plots (BSP) of *Anguis fragilis* lineages distributed in the Balkans. **a** Illyrian-Central European + South Balkan haplogroups together; (**b**) South Balkan haplogroup separately; (**c**) Carniolan + Alpine-Pannonian + North Adriatic (altogether = ‘Slovenian’) haplogroups together

**Fig. 7:**
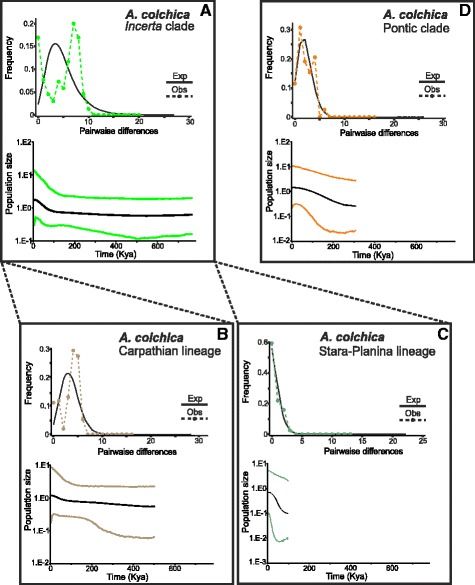
Mismatch distributions (MD) and Bayesian skyline plots (BSP) of *Anguis colchica* lineages distributed in the Balkans. **a** *Incerta* clade; (**b**) Carpathian lineage of the *Incerta* clade; (**c**) Stara-Planina lineage of the *Incerta* clade; (**d**) Pontic clade

**Fig. 8:**
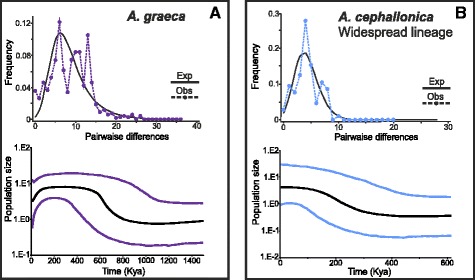
Mismatch distributions (MD) and Bayesian skyline plots (BSP) of *Anguis graeca* (**a**) and *A. cephallonica* (**b**)

The Bayesian skyline plots (BSPs; Figs. 6, 7 and 8) gave evidence of population growth in all tested groups, with the exception of the ‘Slovenian’ populations of *A. fragilis* (Fig. 6c) and the Carpathian populations of *A. colchica*, although a mild and relatively recent (during the last ca 80 Ky) population growth was detected in the Carpathian lineage (Fig. 7b). A sharp population growth was detected in the Stara-Planina lineage of *A. colchica* also since ca 80 Kya (Fig. 7c). Comparing the two main clades of *A. colchica*, population growth started earlier in the Pontic clade (ca 200 Kya; Fig. 7d) than in the *Incerta* clade (80 Kya; Fig. 7a). Considerable population growth was also detected during the last 150 Ky in the ICE + SB haplogroups of *A. fragilis* (Fig. 6a), or since ca 50 Kya when only the SB haplogroup was analysed (Fig. 6b). *Anguis graeca* was analysed as a single population due to its complex genetic variation with many haplogroups. The BSP showed a substantial population growth starting about 700 Kya, the population being stable during the Middle Pleistocene and slightly declining during the last ca 80 Kya (Fig. 8a). In the widespread lineage of *A. cephallonica*, a sign of population growth was detected about 300 Kya ago and the lineage has been stable since the last 100 Kya (Fig. 8b).

The complementary mismatch distributions (MDs; Figs. 6, 7 and 8) showed a ragged distribution of the observed values of pairwise differences in the predominantly Slovenian *A. fragilis* (Fig. 6c), the *Incerta* clade of *A. colchica* (Fig. 7a) and its Carpathian lineage (Fig. 7b), and to some extent also in *A. graeca* and the widespread lineage of *A. cephallonica* (Fig. 8a, b). In the other analysed population groups the observed values mirrored the values expected for a growing- or declining-population model. The neutrality tests showed significant departures from the neutrality in the majority of our groups, except for the predominantly Slovenian haplogroups of *A. fragilis*, the *Incerta* clade of *A. colchica* and its Carpathian lineage (Table 1).

### Genetic diversity and topographic heterogeneity

**Fig. 9:**
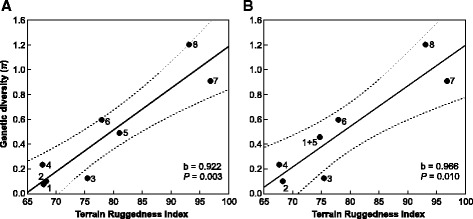
Linear regressions between nucleotide diversity (π) of the Balkan slow-worm evolutionary lineages/haplogroups and modus above the third quartile of the terrain ruggedness index of particular topographic units (mountain systems). In the first analysis (**a**) the Apuseni Mts. were treated as a separate unit, while in the second analysis (**b**) the Apuseni Mts. were considered to be a part of the Carpathians. b - regression coefficient, *P* - probability value. Legends: 1 – Apuseni Mts., 2 – Stara Planina Mts., 3 – Macedonian-Thracian Massif, 4 – Dinarides, 5 – Carpathians, 6 – Prealps, 7 – Peloponnese, 8 – Hellenides (without Peloponnese)

Multiple linear regressions of nucleotide diversity (*π*) of the lineages/haplogroups plotted against the Q3, median above Q3, and modus above Q3 of the terrain ruggedness index (TRI), and an area size inhabited by these lineages/haplogroups, were statistically significant (Table 2). Partial regression analyses, however, revealed that only TRI values, not the area size, had a significant effect on the nucleotide diversity (Table 2, Fig. 9 and Additional file 2: Table S2, Additional file 3: Figure S1 and Additional file 4: Table S3). Standardized (*beta*) regression coefficients were highly significant both when samples from the Apuseni Mts. were included among the Carpathian samples as well as when they were treated separately.

**Table 2:** Results of the multiple linear regressions between nucleotide diversity (*π*), topographic heterogeneity [estimated as the third quartile (Q3) of the terrain ruggedness index (TRI), and median and modus calculated for data above Q3], and the area size of the topographic units inhabited by particular slow-worm lineages/haplogroups

	Apuseni Mts. within the Carpathians		Apuseni Mts. as a separate unit	
	R^2^ */beta*	*P*	R^2^ */beta*	*P*
**TRI (Q3)**/**area size**	**0.814**	**0.035**	**0.830**	**0.012**
**TRI (Q3)**	**0.987**	**0.015**	**0.935**	**0.004**
**area size**	0.274	0.316	0.228	0.281
**TRI (median above Q3)**/**area size**	**0.897**	**0.011**	**0.869**	**0.006**
**TRI (median above Q3)**	**1.110**	**0.004**	**0.973**	**0.002**
**area size**	0.445	0.080	0.299	0.137
**TRI (modus above Q3)**/**area size**	**0.843**	**0.025**	**0.847**	**0.009**
**TRI (modus above Q3)**	**0.966**	**0.010**	**0.922**	**0.003**
**area size**	0.184	0.433	0.079	0.670

In the first set of analyses the Apuseni Mts. were considered to be a part of the Carpathians, in the second set of analyses they were treated as a separate geographical unit. Coefficients of determination (R^2^) were computed for the overall model of multiple regressions [TRI (Q3)/area, TRI (median above Q3)/area, TRI (modus above Q3)/area]. Standardized regression coefficients (*beta*) were calculated for the partial regressions between nucleotide diversity and TRI values, and nucleotide diversity and the area size, respectively

*P* - probability values. Values in bold are statistically significant

## Discussion

### Distribution of slow worms in the Balkans and contact zones

**Fig. 10:**
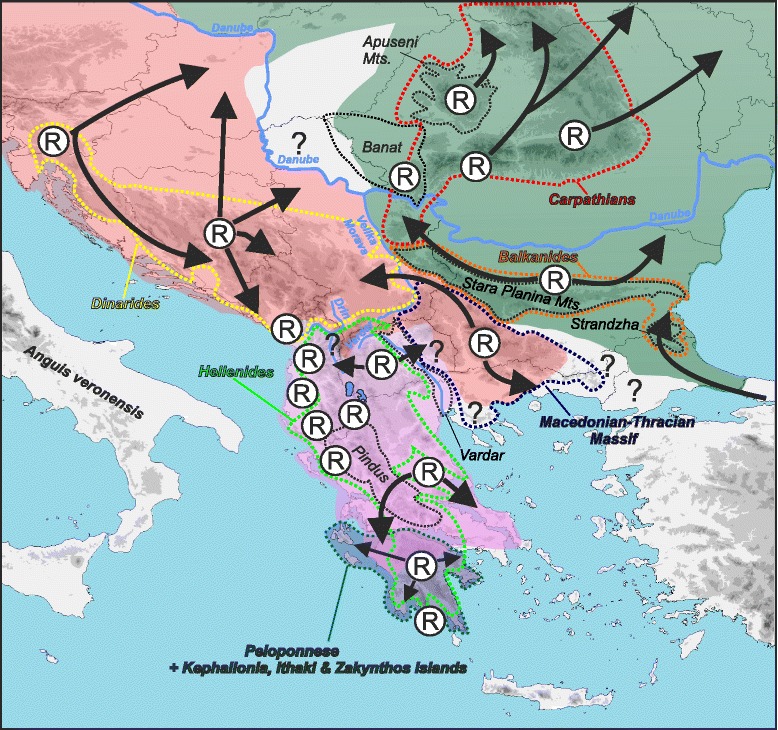
Pleistocene refugia (R) and proposed dispersal postglacial routes of slow worms in the Balkans. Approximate species distributions given in colour shading correspond to the colour code in Fig. 1. Question marks denote missing distribution data

Due to relatively hard-to-interpret morphology and description of several vaguely defined forms and their intermediates in the Balkan Peninsula, the distribution of slow worms remained problematic and conflicting [18, 22, 44]. Recent molecular-phylogenetic studies [14, 15] recognized four species of the genus *Anguis* within the Balkans and have also painted the first coarse-grained picture of their distribution, but the precise ranges have remained to be revealed. Here, based on extensive sampling and molecular identification, we show detailed distribution of all four species inhabiting the Balkan Peninsula (Figs. 1 and 10). The Balkan slow worms are characterized by mostly parapatric distributions, to large extent corresponding with major geomorphological units of the peninsula. We acknowledge that the distribution patterns revealed here may not fully represent species distributions due to the specific characteristics of the used mtDNA marker (maternal and clonal inheritance, reduced effective population size, sex-specific dispersal, relatively common interspecific introgression). However, the overall phylogenetic patterns we found are vastly concordant to previously published ones based on both mtDNA and nuDNA markers [14, 15].

Among our studied species, *Anguis cephallonica* occupies the smallest range limited to the Peloponnese Peninsula and the islands of Kephallonia, Ithaki, and Zakynthos [17, 42, 43]. The distributions of the other three species principally follows the main mountain ranges in the Balkans; *A. fragilis* is distributed in the Dinarides and Macedonian-Thracian Massif, *A. colchica* in the Carpathians, Stara Planina, and Strandzha Mts., and *A. graeca* in the Hellenides.

It appears that while the ranges of *A. fragilis* and *A. graeca* each meet with ranges of two other species in the Balkans (furthermore, *A. fragilis* also forms a contact zone with *A. veronensis* outside the Balkan Peninsula, see [15]), *A. colchica* and *A. cephallonica* only come into contact with one other species. Parts of the contact zones presumably originated by crossing natural barriers such as mountain ridges or river valleys. For instance, the range of *A. graeca* crosses the Vardar River valley and extends from the Hellenides into the Macedonian-Thracian Massif where it forms a contact zone with *A. fragilis*. On the other hand, *A. fragilis* inhabiting predominantly the Dinarides, Macedonian-Thracian Massif, and their vicinity seems to have extended its range to the south, across the northern borderline of the Hellenides, where it forms a contact zone with *A. graeca* (Fig. 1). Historical demographic model indicates that an expansion of the SB haplogroup of *A. fragilis* could probably have happened during the Holocene (Fig. 6b). *Anguis graeca* and *A. cephallonica* form a contact zone and partial sympatry in the northern Peloponnese where they both might have come into contact repeatedly as climatic oscillations and resulting sea-level changes led to repeated connection and disconnection of the peninsula and the mainland during the Pleistocene [17, 45]. It seems that ranges of *A. graeca* and *A. colchica* do not come into recent contact because *A. fragilis* populations are embedded between them.

### Multiple refugia and colonization routes

All four species of slow worms show high levels of intraspecific genetic differentiation in the Balkans and are sub-structured into several divergent lineages or haplogroups. This genetic structure was shaped by local restrictions of ranges into multiple Pleistocene refugia located in the Peloponnese (*A. cephallonica*), Hellenides (*A. graeca*), southern Carpathians (*A. colchica*), and northwestern Dinarides (*A. fragilis*) (Fig. 10). Existence of several smaller and isolated refugia that harboured slow-worm populations during the Pleistocene climatic oscillations within the Balkans is in concordance with the refugia-within-refugia model originally proposed for the Iberian Peninsula [46], and also suggested for the Italian Peninsula based on the phylogeography of *A. veronensis* [15]. This pattern might have more general applicability in the Balkans where multiple refugia were corroborated in both animals e.g. [8, 47–49] and plants e.g. [50, 51]. They are located either in the Mediterranean region (e.g. Adriatic coast, Peloponnese; [52]) or in non-Mediterranean parts of the peninsula (Carpathians, and the Prealps region between the Dinarides and Alps; [4, 5, 53].

The biogeographical histories of slow worms from southern and northern Balkan refugia differ. The ICE haplogroup of *A. fragilis* and several haplogroups of the Carpathian lineage of *A. colchica* colonized broad areas of temperate Europe from their northern extra-Mediterranean refugia. On the contrary, *A. cephallonica*, *A. graeca*, the Pontic and Stara-Planina lineages of *A. colchica*, and the South Balkan haplogroup of *A. fragilis* did not disperse much from their southern Mediterranean refugia and their distribution has remained more localized south of the Danube River (Fig. 10).

In the case of *Anguis fragilis* our results indicate the existence of at least three separate Pleistocene refugia. The South Balkan haplogroup predominantly occurs in the Macedonian-Thracian Massif, where a refugium was presumably located. Outside this mountain range the SB haplogroup only dispersed to the northernmost Hellenides, probably recently, as a common and widespread haplotype was detected there. Populations of the ICE haplogroup colonized vast parts of the western Balkans, but also central and northwestern Europe from a refugium presumably located in the Dinarides. This happened relatively rapidly, which is indicated by (i) a star-like pattern of the haplotype network and low genetic variation of the ICE haplogroup and (ii) the broad area presumably colonized from a single source population [54]. The situation could be vividly illustrated using f1 haplotype (Fig. 2a, c): it is found not only throughout the central and western Balkans, but also in central Europe and as far as the British Isles spread over an area of approximate length of 2000 km [14]. The pattern of the haplotype network and current distribution of *A. fragilis* suggests not only quick expansion to the north, but also a gradual north-to-south/west-to-east expansion during the Pleistocene, which is very rare in terrestrial animals (Fig. 10; [55–57]).

We detected relatively high haplotype diversity of *A. fragilis* in the northern Adriatic region (mainly in Slovenia; Fig. 2b, c). Also the BSP analysis demonstrated population stability for these ‘Slovenian’ haplogroups indicating a long-term survival of slow-worm populations in this region. Such persistence in refugia at foothills of the Alps has been described in several temperate amphibian and reptile species e.g. [58–65]. This region was also probably important in shaping genetic diversity of *A. veronensis,* the species whose main part of the distribution range is located in the Apennine Peninsula [15]. However, the Prealpine slow-worm populations also contributed to the colonization of the Pannonian Basin as indicated by the phylogeographical pattern when extralimital samples were included (Fig. 2a, c; haplotype AF05; [16]).

The Carpathians formed an important extra-Mediterranean refugium of many temperate and cold-adapted species e.g. [40, 56, 66, 67]. This was mainly possible because most of the mountain range remained ice-free during the last glacial maximum [68]. In some taxa, distinct phylogenetic lineages have been detected with distribution restricted to the Carpathians, which indicates their long-term in situ survival (e.g. the newt *Lissotriton vulgaris*, [49, 69]; the toad *Bombina variegata*, [70]). These populations also contributed to the postglacial colonization of Europe. In the Carpathians or their close vicinity we discovered haplotypes of three geographically well-separated lineages of *A. colchica* (Stara-Planina, Banatian, and Carpathian lineages within the *Incerta* clade; Fig. 3). While the Stara-Planina lineage (which is currently also present in the Serbian Carpathians) presumably survived in a refugium outside the Carpathians, the Carpathian and Banatian lineages are together comprised of several haplogroups that could be traced to multiple microrefugia within the Carpathians. Close affinity of these haplotypes (or even identity in some cases, e.g. haplotypes c1, c6) to those from central and north-eastern Europe [14, 15] suggests that these areas were historically colonized from the Carpathian refugia. A very similar colonization pattern of the northern and eastern Europe from the Romanian Carpathians has been described in a rodent *Clethrionomys glareolus* [71].

Despite its limited distribution in the Balkans, the Pontic clade of *A. colchica* shows relatively high mtDNA polymorphism. Close phylogenetic relationships of the southeast Bulgarian and Anatolian populations (own unpublished data) indicate that the Pontic lineage might have colonized the Black Sea region of the Balkans during the Pleistocene when the peninsula was accessible from northern Anatolia via terrestrial route [72, 73].

The Peloponnese, inhabited by endemic *A. cephallonica*, and the region west of the Pindus Mts. (with high haplotype diversity of *A. graeca*) have favourable geography with deep long valleys providing stable climatic conditions. Consequently it is known for high endemism of numerous plants, invertebrates, and vertebrates [17, 74–76]. Multiple refugia in the region have already been proposed [52]. Further in the north, most of Albania and northwestern Greece are surrounded by mountain ranges characterized by steep slopes and deep valleys which could have had a strong isolating effect on *A. graeca* during the Plio-Pleistocene and allowed divergence of its lineages. In contrast, the overall flat Skadar region enabled colonization of southern parts of the region of present-day Montenegro and forming a narrow zone of sympatric occurrence with *A. fragilis*. The existence of several distinct haplogroups in *A. graeca* indicates that this species has a longer and complex evolutionary history. Overall high intraspecific genetic diversity with up to 3.6 % in *p*-distances (Additional file 5: Table S4) suggests older diversification events probably associated with multiple refugia, e.g. in central and southern Albania, northwestern Greece, and northern Peloponnese where the most divergent haplotypes were found.

### Correlation of genetic diversity and topographic heterogeneity

Phylogeographical analysis of all Balkan slow-worm species showed different patterns of intraspecific divergences and genetic diversity for each studied species, presumably mirroring their different, contrasting, evolutionary histories. Specifically, lineages with more pronounced genetic structure inhabit landscapes with higher terrain ruggedness, i.e. higher altitudinal differences, more numerous and deeper valleys, and steeper slopes. Our regression analysis indeed confirms this pattern with high significance – lineages with higher nucleotide diversity inhabit mountain systems characterized by higher elevational differences, i.e. rugged terrain (Table 2, Fig. 9 and Additional file 2: Table S2, Additional file 3: Figure S1 and Additional file 4: Table S3).

The general pattern described as southern richness and northern purity [3] is typical for many taxa on a broad continental scale and can also be observed in slow worms: the species with highest genetic diversity are *A. graeca* and *A. cephallonica* inhabiting the very south of the genus range in the Balkans. A detailed view reveals that even within the relatively small ranges of these species, the highest diversity can be found in smaller and more southerly located areas, corresponding to local microrefugia (or refugia within refugia; [46]). The situation is however different for the two northerly occurring species, *A. colchica* and *A. fragilis*, in which the populations with highest diversity occur in more northerly-located areas in the Balkans. More pronounced altitudinal differences, steep exposed slopes, and generally more heterogeneous landscapes create numerous effective barriers preventing dispersal of small legless lizards, such as slow worms, in which the dispersal ability is also limited by semifossorial lifestyle [77]. Such combination of life history and habitat characteristics provides suitable predispositions for isolation and subsequent divergence of populations. On the other hand, lowlands, plains, low-hill regions and slightly rolling landscapes offer fewer barriers to dispersal and gene flow, and thus divergence occurs less often. Our observations on correlation of slow-worm genetic diversity with topographic ruggedness are fully in concordance with the fact that 33 (63 %) of the 52 identified Mediterranean refugia are situated in submontane and montane areas [52].

## Conclusions

Our study uncovered mitochondrial DNA variation and distribution of four *Anguis* species and hidden diversity of their populations in the Balkans. These species have mostly parapatric distributions that correspond with major mountain ranges. We showed that biogeography of the genus in the Balkans is concordant with the refugia-within-refugia model previously proposed for other main European Peninsulas. The role of Mediterranean as well as extra-Mediterranean refugia was detected in the evolutionary history of slow worms with varying ages and degrees of post-glacial recolonization. Beside climatic historical events, we consider the complex topography of the Balkans as one of the most important factors in shaping recent genetic diversity of slow worms. Topographic heterogeneity seems to be a good predictor of both genetic and species diversity, in general. The pattern observed on slow-worm refugia in the Balkan Peninsula thus illustrates and highlights the fact that many global biodiversity hotspots and endemism centres are located in montane regions [78–82]. As it has been suggested in other taxa [13, 83–86], complex mountain topography offers conditions that could facilitate genetic isolation and divergence and result thus in a high rate of speciation.

### Availability of data and materials

New sequences have been deposited in GenBank (accession numbers KX020147–KX020322) and other input data are provided in Additional file 1: Table S1, Additional file 2: Table S2, Additional file 3: Figure S1, Additional file 4: Table S3 and Additional file 5: Table S4 of this study.

### Ethics

Not applicable.

### Consent to publish.

Not applicable.

## Additional files

Additional file 1: Table S1. A list of samples, their coordinates, locality numbers in maps (Figs. 2, 3, 4 and 5), and GenBank accession numbers. Sample IDs in bold were already used earlier (Gvoždík et al. [14, 15]). (PDF 121 kb) Additional file 2: Table S2. Samples used in regression analyses of nucleotide diversity (π) and terrain ruggedness index (TRI), and their assignment to particular topographic mountain units (mountain ranges). (PDF 27 kb) Additional file 3: Figure S1. A map of demarcated topographic units as defined for regression analyses of nucleotide diversity (π) and terrain ruggedness index (TRI). (PDF 131 kb) Additional file 4: Table S3. Values of nucleotide diversity (π), area size of particular topographic units (in km^2^), and terrain ruggedness index (TRI). The third quartile (TRI Q3), and median (TRI Q3 median) and modus (TRI Q3 modus) of data above TRI Q3 were used in regression analyses. (PDF 21 kb) Additional file 5: Table S4. Average uncorrected *p*-distances calculated among the main evolutionary lineages within each of the four *Anguis* species distributed in the Balkans. The highest values are in bold. (PDF 13 kb)
