# Supplementary material for: Contrasting evolutionary histories of the legless lizards slow worms (Anguis) shaped by the topography of the Balkan Peninsula
Source: BMC Evol Biol. 2016 May 10;16:99. doi: 10.1186/s12862-016-0669-1 (PMC4863322; doi:10.1186/s12862-016-0669-1)
Supplement: Additional file 1: Table S1. — A list of samples, their coordinates, locality numbers in maps (Figs. 2, 3, 4 and 5), and GenBank accession numbers. Sample IDs in bold were already used earlier (Gvoždík et al. [14, 15]). (PDF 121 kb) [file 12862_2016_669_MOESM1_ESM.pdf]

Additional file 1: Table S1. A list of samples, their coordinates, locality numbers in maps (Figs. 2-5), and GenBank accession numbers. Sample IDs in bold were already used earlier (Gvoždík *et al*. 2010, 2013).

Species	lineage/haplogroup	Locality	Coordinates		Sample ID	Locality number	Published haplotype	GenBank accession number	References
			N	E					
Anguis cephallonica		**Greece**							
	Widespread lineage	Achladocampos, Ktenia Mts.	37.51	22.61	-	184	-	KJ634783	Thanou *et al.*, 2014
	Widespread lineage	Akoli Lake, Kephallonia Isl.	38.19	20.67	-	173	-	KJ634790	Thanou *et al.*, 2014
	Widespread lineage	Akoli Lake, Kephallonia Isl.	38.19	20.67	-	173	-	KJ634791	Thanou *et al.*, 2014
	Widespread lineage	Dirrachio, Erymanthos Mts.	37.15	22.19	-	181	-	KJ634793	Thanou *et al.*, 2014
	Widespread lineage	Elati, Mainalo Mts.	37.61	22.15	-	179	-	KJ634782	Thanou *et al.*, 2014
	Widespread lineage	Gialova	36.95	21.70	**A085gr**	186	**ce1**	FJ666586	Gvoždík *et al.*, 2010, 2013
	Widespread lineage	Laggadia, Mainalo Mts.	37.67	22.02	-	177	-	KJ634788	Thanou *et al.*, 2014
	Mani lineage	Lagia, Mani Pen.	36.47	22.47	-	185	-	KJ634795	Thanou *et al.*, 2014
	Widespread lineage	Leontari, Taygetos Mts.	37.31	22.15	-	183	**ce1**	KJ634792	Thanou *et al.*, 2014
	Widespread lineage	Neochori, Taygetos Mts.	37.16	22.25	-	182	-	KJ634794	Thanou *et al.*, 2014
	Widespread lineage	Pirgaki, Mainalo Mts.	37.63	22.15	-	180	-	KJ634786	Thanou *et al.*, 2014
	Widespread lineage	Rodia, Kyparissia	37.21	21.73	-	187	-	KJ634785	Thanou *et al.*, 2014
	Widespread lineage	Salmeniko, Ziria Mts.	38.27	21.95	-	174	-	KJ634784	Thanou *et al.*, 2014
	Widespread lineage	Stymfalia Lake	37.88	22.48	**A048gr**	176	**ce2**	FJ666587	Gvoždík *et*

									al., 2010, 2013
	Widespread lineage	Tripotama, Erymanthos Mts.	37.86	21.89	-	175	-	KJ634789	Thanou *et al*., 2014
	Widespread lineage	Valtesiniko, Mainalo Mts.	37.68	22.11	-	178	-	KJ634787	Thanou *et al*., 2014
Anguis colchica		**Bulgaria**							
	Stara-Planina lineage	Bozhenitsa	43.00	23.80	Abg33	117	-	KX020147	This study
	Pontic clade	Brodilovo pass	42.10	27.83	Abg46	131	-	KX020148	This study
	Stara-Planina lineage	Garvan	44.11	26.89	Abg29	127	-	KX020149	This study
	Stara-Planina lineage	Godech	43.01	23.05	Abg41	116	-	KX020150	This study
	Pontic clade	Gramatikovo	42.03	27.63	Abg49	136	-	KX020151	This study
	Pontic clade	Izgrev	42.12	27.76	Abg47	133	-	KX020152	This study
	Pontic clade	Kondolovo	42.09	27.65	Abg48	135	-	KX020153	This study
	Stara-Planina lineage	Letnitsa	43.31	25.13	Abg36	121	-	KX020154	This study
	Stara-Planina lineage	Makotsevo	42.69	23.80	Abg23	119	-	KX020155	This study
	Pontic clade	Mladežko	42.16	27.43	Abg53	138	-	KX020156	This study
	Pontic clade	Park Rosenec	42.43	27.53	Abg54	129	-	KX020157	This study
	Pontic clade	Pass to Slivarovo	41.98	27.57	Abg50	137	-	KX020158	This study
	Pontic clade	Pass to Slivarovo	41.98	27.57	Abg51	137	-	KX020159	This study
	Stara-Planina lineage	Patresh	43.31	25.34	Abg37	122	-	KX020160	This study
	Stara-Planina lineage	Pravetz	42.89	23.91	Abg10	118	-	KX020161	This study
	Stara-Planina lineage	Pravetz	42.89	23.91	Abg11	118	-	KX020162	This study
	Pontic clade	Ropotamo	42.30	27.72	Abg13	130	-	KX020163	This study
	Stara-Planina lineage	Shipka	42.75	25.31	Abg05	124	-	KX020164	This study
	Stara-Planina lineage	Shipka	42.75	25.31	Abg06	124	-	KX020165	This study
	Stara-Planina lineage	Shipka 2	42.76	25.31	Abg30	123	-	KX020166	This study
	Stara-Planina lineage	Shumen	43.26	26.89	Abg12	126	-	KX020167	This study
	Stara-Planina lineage	Sinagovtsi	43.89	22.75	Abg04	113	-	KX020168	This study
	Pontic clade	Sinemorec	42.06	27.97	Abg01	132	-	KX020169	This study
	Pontic clade	Sinemorec	42.06	27.97	Abg02	132	-	KX020170	This study
	Pontic clade	Sinemorec	42.06	27.97	Abg03	132	-	KX020171	This study
	Stara-Planina lineage	Slavyani	43.27	24.65	Abg24	120	-	KX020172	This study
	Stara-Planina lineage	Slavyani	43.27	24.65	Abg25	120	-	KX020173	This study
	Stara-Planina lineage	Srebarna	44.07	27.04	Abg28	128	-	KX020174	This study
	Pontic clade	Strandža (Silkosia)	42.08	27.74	Abg35	134	-	KX020175	This study
	Stara-Planina lineage	Targovishte	43.54	22.74	Abg43	114	-	KX020176	This study
	Stara-Planina lineage	Triyavna	42.86	25.48	Abg21	125	-	KX020177	This study
		**Romania**							
	Carpathian lineage IV	Bazna	46.20	24.28	Aro02	100	**c6, c12**	KX020178	This study
	Banatian lineage	Cheile Sohodolului	45.14	23.13	Aro12	108	-	KX020179	This study

	Carpathian lineage II	Cheile Sohodolului	45.14	23.13	Aro13	108	-	KX020180	This study
	Carpathian lineage I	Finatale Clujuluj	46.83	23.62	**A118ro**	96	**c1, c2, c5**	FJ666580	Gvoždík *et al*., 2010, 2013
	Carpathian lineage I	Geoagiu de Sus	46.30	23.52	Aro07	99	**c1, c2, c5**	KX020181	This study
	Carpathian lineage I	Geoagiu de Sus 2	46.29	23.54	Aro08	98	**c1, c2, c5**	KX020182	This study
	Carpathian lineage I	Huta	47.00	22.94	Aro03	95		KX020183	This study
	Carpathian lineage I	Huta	47.00	22.94	Aro04	95		KX020184	This study
	Carpathian lineage III	Laslea	46.19	24.64	Aro11	101	-	KX020185	This study
	Carpathian lineage IV	Maramures Mts.	47.86	24.15	Aro18	93	**c6, c12**	KX020186	This study
	Carpathian lineage III	Nou Sasesc	46.11	24.59	Aro10	102	-	KX020187	This study
	Carpathian lineage III	Poieni	47.04	27.69	Aro19	105	-	KX020188	This study
	Carpathian lineage I	Runcu Salvei	47.20	24.20	Aro01	94	-	KX020189	This study
	Carpathian lineage III	Sacadat	46.61	25.06	Aro09	103	-	KX020190	This study
	Carpathian lineage IV	Schitul Locurele	45.25	23.37	Aro16	106	**c6, c12**	KX020191	This study
	Stara-Planina lineage	Sfanta Elena	44.67	21.71	Aro14	111	-	KX020192	This study
	Stara-Planina lineage	Sfanta Elena	44.67	21.71	Aro15	111	-	KX020193	This study
	Carpathian lineage III	Subcetate	46.42	25.40	Aro05	104	-	KX020194	This study
	Carpathian lineage IV	Subcetate	46.42	25.40	Aro06	104	**c6, c12**	KX020195	This study
	Carpathian lineage II	Valea Bratcu	45.25	23.34	Aro17	107	-	KX020196	This study
	Carpathian lineage I	Vartop, Bihar Mts.	46.51	22.66	AC01	97	**c1, c2, c5**	KFT736829	Szabó & Vörös, 2014
		**Serbia**							
	Stara-Planina lineage	Grza	43.90	21.65	gd28rs	112	-	KX020197	This study
	Banatian lineage	Jasenovo	44.92	21.28	gd13rs	110	-	KX020198	This study
	Stara-Planina lineage	Ponor, Stara Mts.	43.25	22.80	gd29rs	115	-	KX020199	This study
	Banatian lineage	Vršački breg	45.13	21.35	gd10rs	109	-	KX020200	This study
Anguis fragilis		**Albania**							
	Illyrian-Central European	Nikç	42.47	19.67	Aal01	62	-	KX020201	This study
		**Bosnia and Herzegovina**							
	Illyrian-Central European	Gornji Podgradci	45.04	16.91	Aba01	17	**f1, f2, f3, f12, f13, AF01**	KX020202	This study
	Illyrian-Central European	Kordići	43.96	17.46	Aba05	20	**f1, f2, f3, f12, f13, AF01**	KX020203	This study
	Illyrian-Central European	Korita	43.03	18.49	**Aba18**	19	**f11**	KC881542	Gvoždík et al. 2013
	Illyrian-Central European	Maglić Mt.	43.28	18.71	Aba09	29	**f1, f2, f3, f12, f13, AF01**	KX020204	This study
	Illyrian-Central European	Maglić Mt.	43.28	18.71	Aba10	29	**f1, f2, f3, f12, f13, AF01**	KX020205	This study
	Illyrian-Central	Nevesinje	43.24	18.09	Aba06	25	**f1, f2, f3, f12, f13,**	KX020206	This study

	European						**AF01**		
	Illyrian-Central European	Nišići	44.05	18.46	Aba11	23	**f1, f2, f3, f12, f13, AF01**	KX020207	This study
	Illyrian-Central European	Nišići	44.05	18.46	Aba12	23	**f1, f2, f3, f12, f13, AF01**	KX020208	This study
	Illyrian-Central European	Nišići	44.05	18.46	Aba13	23	**f1, f2, f3, f12, f13, AF01**	KX020209	This study
	Illyrian-Central European	Oštrelj	44.47	16.40	Aba02	14	**f1, f2, f3, f12, f13, AF01**	KX020210	This study
	Illyrian-Central European	Oštrelj	44.47	16.40	Aba03	14		KX020211	This study
	Illyrian-Central European	Oštrelj	44.47	16.40	Aba04	14	**f1, f2, f3, f12, f13, AF01**	KX020212	This study
	Illyrian-Central European	Požarnica	44.53	18.77	Aba14	22	**f1, f2, f3, f12, f13, AF01**	KX020213	This study
	Illyrian-Central European	Požarnica	44.53	18.77	Aba15	22	**f1, f2, f3, f12, f13, AF01**	KX020214	This study
	Illyrian-Central European	Suha	43.30	18.65	Aba08	26		KX020215	This study
	Illyrian-Central European	Suha 2	43.30	18.66	Aba19	27	**f f1, f2, f3, f12, f13, AF01**	KX020216	This study
	Illyrian-Central European	Tjentište	43.36	18.70	Aba07	28	**f10**	KX020217	This study
	Illyrian-Central European	Trnovo	43.66	18.44	Aba16	24	**f1, f2, f3, f12, f13, AF01**	KX020218	This study
	Illyrian-Central European	Trnovo	43.66	18.44	Aba17	24	**f1, f2, f3, f12, f13, AF01**	KX020219	This study
		**Bulgaria**							
	South Balkan	Aleko, Vitosha Mts.	42.59	23.28	Abg22	73	-	KX020220	This study
	South Balkan	Aleko, Vitosha Mts.	42.59	23.28	Abg26	73	-	KX020221	This study
	South Balkan	Asenovgrad	41.98	24.87	Abg44	92	-	KX020222	This study
	South Balkan	Belasitsa Mts.	41.35	23.12	Abg34	82	**f5**	KX020223	This study
	South Balkan	Belasitsa Mts. 2	41.32	23.12	Abg38	83	**f5**	KX020224	This study
	South Balkan	Kirilova Polyana	42.15	23.39	Abg17	79	-	KX020225	This study
	South Balkan	Krusha	42.89	22.78	Abg40	68	-	KX020226	This study
	South Balkan	Lozenska Mts.	42.58	23.44	Abg18	75	-	KX020227	This study
	South Balkan	Lozenska Mts.	42.58	23.44	Abg19	75	-	KX020228	This study
	South Balkan	Lozenska Mts. 2	42.59	23.43	Abg20	74	-	KX020229	This study
	South Balkan	Novo Selo	42.17	22.68	Abg16	72	**f5**	KX020230	This study
	South Balkan	Osogovska Mts.	42.17	22.62	Abg27	71	**f5**	KX020231	This study
	South Balkan	Persenk	41.81	24.54	Abg39	91	-	KX020232	This study
	South Balkan	Sapareva Banya	42.24	23.31	Abg08	77	-	KX020233	This study
	South Balkan	Sapareva Banya 2	42.26	23.28	Abg09	78	**f5**	KX020234	This study
	South Balkan	Slavyanka	41.39	23.60	Abg15	84	**f5**	KX020235	This study
	South Balkan	Tsrancha	41.56	24.09	Abg31	86	**f5**	KX020236	This study

	South Balkan	Varvara	42.13	24.12	Abg45	85	-	KX020237	This study
	South Balkan	Yavorov	41.85	23.40	Abg32	80	-	KX020238	This study
	South Balkan	Železnica, Vitosha Mts.	42.53	23.35	Abg42	76	-	KX020239	This study
	South Balkan	Zhablyano	42.49	22.80	Abg07	69	-	KX020240	This study
	South Balkan	Zhilentsi	42.25	22.63	Abg14	70	**f5**	KX020241	This study
		**Croatia**							
	Illyrian-Central European	Begovo Razdolje	45.30	14.91	Ahr10	8	**f1, f2, f3, f12, f13, AF01**	KX020242	This study
	Illyrian-Central European	Dinara	44.04	16.41	Ahr06	15	**f1, f2, f3, f12, f13, AF01**	KX020243	This study
	Illyrian-Central European	Hrvatska Dubica	45.18	16.80	Ahr08	16	-	KX020244	This study
	Illyrian-Central European	Kamešnica	43.71	16.88	Ahr05	18	**f1, f2, f3, f12, f13, AF01**	KX020245	This study
	Illyrian-Central European	Medvedica	45.89	16.03	Ahr09	9	**f1, f2, f3, f12, f13, AF01**	KX020246	This study
	North Adriatic	Njivice	45.16	14.54	Ahr13	7	-	KX020247	This study
	Illyrian-Central European	Poštak	44.25	16.11	Ahr07	13	**f1, f2, f3, f12, f13, AF01**	KX020248	This study
	Illyrian-Central European	Prezid	44.24	15.80	**Ahr01**	12	**f1, f2, f3, f12, f13**	FJ666554	Gvoždík et al. 2013
	Illyrian-Central European	Prezid	44.24	15.80	Ahr02	12	**f1, f2, f3, f12, f13, AF01**	KX020249	This study
	Illyrian-Central European	Prezid	44.24	15.80	Ahr03	12	**f1, f2, f3, f12, f13**	KX020250	This study
	Illyrian-Central European	Ramići	44.34	15.48	Ahr11	11	**f1, f2, f3, f12, f13, AF01**	KX020251	This study
	Illyrian-Central European	Spačva	45.04	18.90	Ahr12	21	**f1, f2, f3, f12, f13**	KX020252	This study
	Illyrian-Central European	Vugrovec	45.89	16.04	Ahr04	10	**f1, f2, f3, f12, f13, AF01**	KX020253	This study
		**Greece**							
	South Balkan	Lepida	41.37	24.63	**A093gr**	90	**f5**	FJ666558	Gvoždík *et al.*, 2010, 2013
	South Balkan	Livadero	41.30	24.21	Agr01	88	**f5**	KX020254	This study
	South Balkan	Mesoropi	40.89	24.06	**A052gr**	89	**f4**	FJ666557	Gvoždík *et al.*, 2010, 2013
	South Balkan	Skaloti	41.45	24.31	A094gr	87	**f5**	KX020255	This study
		**Montenegro**							
	Illyrian-Central European	Ada Bojana	41.85	19.35	gd44me	61	-	KX020256	This study
	Illyrian-Central	Boljevići	42.22	19.08	gd46me	59	**f1, f2, f3, f12, f13,**	KX020257	This study

	European						**AF01**		
	Illyrian-Central European	Cetinje	42.39	18.91	A216me	53	-	KX020258	This study
	Illyrian-Central European	Cetinje 2	42.39	18.92	A137me	54	-	KX020259	This study
	Illyrian-Central European	Dobrota	42.45	18.77	gd23me	47	-	KX020260	This study
	Illyrian-Central European	Ivanova Korita	42.37	18.83	gd39me	49	-	KX020261	This study
	Illyrian-Central European	Jablan	42.62	19.43	gd50me	60	-	KX020262	This study
	Illyrian-Central European	Koštanjica	42.47	18.65	gd24me	44	-	KX020263	This study
	Illyrian-Central European	Krtoli	42.40	18.68	gd25me	45	-	KX020264	This study
	Illyrian-Central European	Lokve Martiničke	42.58	19.23	gd48me	56	**f1, f2, f3, f12, f13, AF01**	KX020265	This study
	Illyrian-Central European	Lovćen	42.39	18.84	Ame04	51	**f1, f2, f3, f12, f13, AF01**	KX020266	This study
	Illyrian-Central European	Njeguši	42.43	18.85	gd42me	50	-	KX020267	This study
	Illyrian-Central European	Orjen	42.52	18.53	Ame03	42	-	KX020268	This study
	Illyrian-Central European	Resna	42.50	18.88	gd41me	48	-	KX020269	This study
	Illyrian-Central European	Šišici	42.37	18.78	gd40me	46	-	KX020270	This study
	Illyrian-Central European	Stijena	42.52	19.25	gd49me	57	-	KX020271	This study
	Illyrian-Central European	Sutomore	42.14	19.04	Ame02	58	-	KX020272	This study
	Illyrian-Central European	Suva Ponikvica	42.67	19.25	gd47me	55	-	KX020273	This study
	Illyrian-Central European	Sveti Stefan	42.25	18.88	gd26me	52	**f1, f2, f3, f12, f13, AF01**	KX020274	This study
	Illyrian-Central European	Ubli	42.52	18.63	gd35me	43	-	KX020275	This study
		**Republic of Macedonia**							
	South Balkan	Pečkovo	41.78	20.83	Amk02	63	**f5**	KX020276	This study
	South Balkan	Prevedena	41.59	22.87	Amk01	81	**f5**	KX020277	This study
		**Serbia**							
	Illyrian-Central European	Avala	44.68	20.52	gd17rs	34	**f1, f2, f3, f12, f13, AF01**	KX020278	This study
	Illyrian-Central European	Avala 2	44.68	20.51	Ars12	35	-	KX020279	This study

	South Balkan	Brod	42.87	22.29	Ars03	66	**f5**	KX020280	This study
	South Balkan	Dimitrovgrad	43.02	22.78	gd31rs	67	-	KX020281	This study
	Illyrian-Central European	Fruška Gora	45.11	19.78	Ars13	32	**f1, f2, f3, f12, f13, AF01**	KX020282	This study
	Illyrian-Central European	Fruška Gora 2	45.15	19.70	Ars14	30	-	KX020283	This study
	Illyrian-Central European	Gornja Trešnjica	44.12	19.50	gd27rs	39	**f10**	KX020284	This study
	Illyrian-Central European	Kaludjerica	44.75	20.55	gd16rs	36	**f1, f2, f3, f12, f13, AF01**	KX020285	This study
	Illyrian-Central European	Kaludjerske Bare	43.96	19.41	Ars01	40	**f10**	KX020286	This study
	Illyrian-Central European	Kaludjerske Bare	43.96	19.41	Ars02	40	**f10**	KX020287	This study
	South Balkan	Kriva Feja	42.58	22.13	gd32rs	64	**f5**	KX020288	This study
	Illyrian-Central European	Novi Vitojevci	44.78	19.80	gd22rs	33	-	KX020289	This study
	Illyrian-Central European	Petnica	44.24	19.93	Ars09	38	**f10**	KX020290	This study
	Illyrian-Central European	Petnica	44.24	19.93	Ars10	38	**f10**	KX020291	This study
	Illyrian-Central European	Petnica	44.24	19.93	Ars11	38	-	KX020292	This study
	Illyrian-Central European	Rogača	44.45	20.52	gd18rs	37	**f1, f2, f3, f12, f13, AF01**	KX020293	This study
	South Balkan	Ruplje	42.83	22.21	Ars15	65	**f5**	KX020294	This study
	Illyrian-Central European	Sremska Kamenica	45.21	19.84	Ars04	31	**f1, f2, f3, f12, f13, AF01**	KX020295	This study
	Illyrian-Central European	Sremska Kamenica	45.21	19.84	Ars05	31	**f1, f2, f3, f12, f13**	KX020296	This study
	Illyrian-Central European	Sremska Kamenica	45.21	19.84	Ars06	31	**f1, f2, f3, f12, f13, AF01**	KX020297	This study
	Illyrian-Central European	Sremska Kamenica	45.21	19.84	Ars07	31	**f1, f2, f3, f12, f13, AF01**	KX020298	This study
	Illyrian-Central European	Sremska Kamenica	45.21	19.84	Ars08	31	**f1, f2, f3, f12, f13, AF01**	KX020299	This study
	Illyrian-Central European	Užice	43.86	19.84	**A043Brs**	41	**f10**	KC881541	Gvoždík *et al.*, 2010, 2013
		**Slovenia**							
	Alpine-Pannonian	Bohinj Lake	46.29	13.90	**A065si**	3	**f6**	FJ666559	Gvoždík *et al.*, 2010, 2013
	North Adriatic	Dobrava	45.52	13.62	gd06si	1	**AF06**	KX020300	This study
	North Adriatic	Fiesa, Piran	45.52	13.58	**AF06**	2	**AF06**	KF736836	Szabó & Vörös, 2014

	Carniolan	Jablanica	45.53	14.28	gd09si	6	-	KX020301	This study
	Carniolan	Kozina	45.60	13.95	**A205si**	5	**f9**	KC881540	Gvoždík et al. 2013
	Alpine-Pannonian	Zalošče	45.90	13.90	**A206si**	4	**f6**	FJ666559	Gvoždík et al. 2013
Anguis graeca		**Albania**							
	graeca XII	Dajti Mt.	41.36	19.91	A154al	141	-	KX020302	This study
	graeca IX	Diviakë	40.95	19.47	**A029al**	142	**g13**	FJ666572	Gvoždík *et al.*, 2010, 2013
	graeca III	Diviakë	40.95	19.47	A027al	142	-	KX020303	This study
	graeca V	Dukat	40.21	19.58	**A023al**	143	**g15**	FJ666574	Gvoždík *et al.*, 2010, 2013
	graeca V	Dukat	40.21	19.58	A024al	143	-	KX020304	This study
	graeca IV	Ersekë	40.32	20.67	**A025al**	156	**g4**	FJ666563	Gvoždík *et al.*, 2010, 2013
	graeca IV	Ersekë	40.32	20.67	**A044al**	156	**g5**	FJ666564	Gvoždík *et al.*, 2010, 2013
	graeca IV	Ersekë	40.32	20.67	A045al	156	**g5**	KX020305	This study
	graeca II	Himarë	40.10	19.75	**A028al**	144	**g7**	FJ666566	Gvoždík *et al.*, 2010, 2013
	graeca VII	Korcë	40.61	20.82	**A022al**	157	**g16**	FJ666575	Gvoždík *et al.*, 2010, 2013
	graeca X	Milot	41.69	19.74	Aal02	140	-	KX020306	This study
	graeca II	Syri i Kaltër	39.92	20.19	**A026Bal**	145	**g8**	FJ666567	Gvoždík *et al.*, 2010, 2013
		**Greece**							
	graeca I	Ag. Vasilios, Patra	38.31	21.80	-	166	**g1**	KJ634797	Thanou *et al.*, 2014
	graeca V	Ampelochori	39.53	21.03	**A092gr**	161	**g10**	FJ666569	Gvoždík *et al.*, 2010, 2013
	graeca IV	Aoos River	40.05	20.76	**A091gr**	155	**g6**	FJ666565	Gvoždík *et al.*, 2010, 2013
	KJ634800	Doxa Lake, Feneos	37.92	22.29	-	172	-	KJ634800	Thanou *et al.*, 2014
	KJ634801	Doxa Lake, Feneos	37.92	22.29	-	172	-	KJ634801	Thanou *et al.*, 2014
	graeca I	Fylakti	39.30	21.68	**A089gr**	163	**g2**	FJ666561	Gvoždík *et*

									al., 2010, 2013
	graeca V	Gliki	39.33	20.55	**A053gr**	154	**g9**	FJ666568	Gvoždík *et al*., 2010, 2013
	graeca VI	Gliki	39.33	20.55	**A054gr**	154	**g12**	FJ666571	Gvoždík *et al*., 2010, 2013
	graeca V	Kerkyra - Chrisida	39.59	19.90	**A095gr**	147	**g11**	FJ666570	Gvoždík *et al*., 2010, 2013
	graeca V	Kerkyra - Gardiki	39.48	19.88	A238gr	149	-	KX020307	This study
	graeca V	Kerkyra - Moraitika	39.48	19.92	A246gr	150	**g9**	KX020308	This study
	graeca V	Kerkyra - Perivoli	39.41	20.02	A239gr	151	-	KX020309	This study
	graeca V	Kerkyra - Perivoli 2	39.41	20.01	A240gr	153	-	KX020310	This study
	graeca V	Kerkyra - Perivoli 2	39.41	20.01	A241gr	153	-	KX020311	This study
	Albanian-Greek	Kerkyra - Poulades	39.67	19.77	A243gr	146	-	KX020312	This study
	graeca V	Kerkyra - Stavros	39.53	19.91	A247gr	148	-	KX020313	This study
	graeca V	Kerkyra - Stavros	39.53	19.91	A248gr	148	-	KX020314	This study
	graeca V	Kerkyra - Vitalades	39.41	20.02	A242gr	152	-	KX020315	This study
	Greek	Kremasta Lake, Karpenisi	38.88	21.49	-	164	-	KJ634796	Thanou *et al*., 2014
	graeca I	Kryoneritis	38.93	23.28	**A086gr**	171	**g2**	FJ666561	Gvoždík *et al*., 2010, 2013
	graeca I	Mornos River	38.49	22.06	**A049gr**	168	**g1**	FJ666560	Gvoždík *et al*., 2010, 2013
	graeca I	Mornos River	38.49	22.06	**A050gr**	168	**g1**	FJ666560	Gvoždík *et al*., 2010, 2013
	graeca I	Mornos River	38.49	22.06	**A051gr**	168	**g1**	FJ666560	Gvoždík *et al*., 2010, 2013
	graeca I	Pefki-Artemision	39.01	23.23	**A087gr**	170	**g2**	FJ666561	Gvoždík *et al*., 2010, 2013
	graeca I	Pefki-Artemision	39.01	23.23	**A088gr**	170	**g2**	FJ666561	Gvoždík *et al*., 2010, 2013
	graeca I	Pertouli	39.54	21.47	**A090gr**	162	**g2**	FJ666561	Gvoždík *et al*., 2010, 2013
	graeca I	Stomio	39.89	22.62	**A076gr**	169	**g3**	FJ666562	Gvoždík *et al*., 2010, 2013

	graeca I	Stomio	39.89	22.62	A077gr	169	**g3**	KX020316	This study
	graeca I	Stomio	39.89	22.62	A078gr	169	**g2**	KX020317	This study
	graeca I	Strofylia Lake, Pyrgos	38.15	21.40	KJ634799	165	-	KJ634799	Thanou *et al.*, 2014
	graeca I	Velvina, Nefpaktos	38.40	21.78	KJ634798	167	-	KJ634798	Thanou *et al.*, 2014
		**Montenegro**					-		
	graeca VIII	Ulcinj	41.93	19.21	**A064me**	139	**g14b**	FJ666573	Gvoždík *et al.*, 2010, 2013
		**Republic of Macedonia**							
	graeca XI	Bistra Mts.	41.53	20.66	Amk04	158	-	KX020318	This study
	graeca XI	Bistra Mts.	41.53	20.66	Amk05	158	-	KX020319	This study
	graeca XI	Bistra Mts.	41.53	20.66	Amk06	158	-	KX020320	This study
	graeca XI	Kriva Palanka	42.20	22.31	Amk07	160	-	KX020321	This study
	graeca XI	Vrbjani	41.33	21.38	Amk03	159	-	KX020322	This study

References:

Gvoždík V, Benkovský N, Crottini A, Bellati A, Moravec J, Romano A, Sacchi R, Jandzik D. An ancient lineage of slow worms, genus *Anguis* (Squamata: Anguidae), survived in the Italian Peninsula. Mol. Phylogenet. Evol. 2013; 69:1077-92.

Gvoždík V, Jandzik D, Lymberakis P, Jablonski D, Moravec J. Slow Worm, *Anguis fragilis* (Reptilia: Anguidae) as a species complex: Genetic structure reveals deep divergences. Mol Phylogenet Evol. 2010;55:460-472.

Szabó K, Vörös J. Distribution and hybridization of *Anguis fragilis* and *A. colchica* in Hungary. Amphibia-Reptilia. 2014;35:135-140.

Thanou E, Giokas S, Kornilios P. Phylogeography and genetic structure of the slow worms *Anguis cephallonica* and *Anguis graeca* (Squamata: Anguidae) from the southern Balkan Peninsula. Amphibia-Reptilia. 2014;35:263-269.
