# Supplementary material for: Contrasting evolutionary histories of the legless lizards slow worms (Anguis) shaped by the topography of the Balkan Peninsula
Source: BMC Evol Biol. 2016 May 10;16:99. doi: 10.1186/s12862-016-0669-1 (PMC4863322; doi:10.1186/s12862-016-0669-1)
Supplement: Additional file 2: Table S2. — Samples used in regression analyses of nucleotide diversity (π) and terrain ruggedness index (TRI), and their assignment to particular topographic mountain units (mountain ranges). (PDF 27 kb) [file 12862_2016_669_MOESM2_ESM.pdf]

Additional file 2: Table S2. Samples used in regression analyses of nucleotide diversity (π) and terrain ruggedness index (TRI), and their assignment to the particular topographic mountain units (mountain ranges).

Mountain unit	Locality number in Figs. 2-5	Sample ID
Apuseni Mts.	95	Aro03, Aro04
Apuseni Mts.	96	A118ro
Apuseni Mts.	97	AC01
Apuseni Mts.	98	Aro08
Apuseni Mts.	99	Aro07
Carpathians	93	Aro18
Carpathians	100	Aro02
Carpathians	101	Aro11
Carpathians	102	Aro10
Carpathians	103	Aro09
Carpathians	104	Aro05, Aro06
Carpathians	106	Aro16
Carpathians	107	Aro17
Carpathians	108	Aro13
Dinarides	8	Ahr10
Dinarides	11	Ahr11
Dinarides	12	Ahr02, Ahr03
Dinarides	13	Ahr07
Dinarides	14	Aba02, Aba03, Aba04
Dinarides	15	Ahr06
Dinarides	18	Ahr05
Dinarides	20	Aba05
Dinarides	22	Aba14, Aba15
Dinarides	23	Aba11, Aba12, Aba13
Dinarides	24	Aba16, Aba17
Dinarides	25	Aba06
Dinarides	19	Aba18
Dinarides	26	Aba08
Dinarides	27	Aba19
Dinarides	28	Aba07
Dinarides	29	Aba09, Aba10
Dinarides	39	gd27rs
Dinarides	38	Ars09, Ars10, Ars11
Dinarides	40	Ars01, Ars02

Dinarides	41	A043Brs
Dinarides	42	Ame03
Dinarides	43	gd35me
Dinarides	44	gd24me
Dinarides	45	gd25me
Dinarides	46	gd40me
Dinarides	47	gd23me
Dinarides	48	gd41me
Dinarides	49	gd39me
Dinarides	50	gd42me
Dinarides	51	Ame04
Dinarides	52	gd26me
Dinarides	53	A216me
Dinarides	54	A137me
Dinarides	55	gd47me
Dinarides	56	gd48me
Dinarides	57	gd49me
Dinarides	58	Ame02
Dinarides	59	gd46me
Dinarides	60	gd50me
Dinarides	61	gd44me
Dinarides	62	Aal01
Hellenides	140	Aal02
Hellenides	141	A154al
Hellenides	143	A023al
Hellenides	144	A028al
Hellenides	145	A026Bal
Hellenides	154	A053gr, A054gr
Hellenides	155	A091gr
Hellenides	156	A025al, A044al, A045al
Hellenides	157	A022al
Hellenides	158	Amk04, Amk05, Amk06
Hellenides	161	A092gr
Hellenides	162	A090gr
Hellenides	163	A089gr
Hellenides	164	KJ634796
Hellenides	167	KJ634798
Hellenides	168	A049gr, A050gr, A051gr
Prealps	3	A065si
Prealps	4	A206si
Prealps	5	A205si
Prealps	6	gd09si
Peloponnese	174	KJ634784

Peloponnese	175	KJ634789
Peloponnese	176	A048gr
Peloponnese	177	KJ634788
Peloponnese	178	KJ634787
Peloponnese	179	KJ634782
Peloponnese	180	KJ634786
Peloponnese	181	KJ634793
Peloponnese	182	KJ634794
Peloponnese	183	KJ634792
Peloponnese	184	KJ634783
Peloponnese	185	KJ634795
Macedonian-Thracian Massif	64	gd32rs
Macedonian-Thracian Massif	65	Ars15
Macedonian-Thracian Massif	66	Ars03
Macedonian-Thracian Massif	67	gd31rs
Macedonian-Thracian Massif	68	Abg40
Macedonian-Thracian Massif	69	Abg07
Macedonian-Thracian Massif	70	Abg14
Macedonian-Thracian Massif	71	Abg27
Macedonian-Thracian Massif	72	Abg16
Macedonian-Thracian Massif	73	Abg22, Abg26
Macedonian-Thracian Massif	74	Abg20
Macedonian-Thracian Massif	75	Abg18, Abg19
Macedonian-Thracian Massif	76	Abg42
Macedonian-Thracian Massif	77	Abg08
Macedonian-Thracian Massif	78	Abg09
Macedonian-Thracian Massif	79	Abg17
Macedonian-Thracian Massif	80	Abg32
Macedonian-Thracian Massif	81	Amk01
Macedonian-Thracian Massif	82	Abg34

Macedonian-Thracian Massif	83	Abg38
Macedonian-Thracian Massif	84	Abg15
Macedonian-Thracian Massif	85	Abg45
Macedonian-Thracian Massif	86	Abg31
Macedonian-Thracian Massif	87	A094gr
Macedonian-Thracian Massif	88	Agr01
Macedonian-Thracian Massif	90	A093gr
Macedonian-Thracian Massif	91	Abg39
Macedonian-Thracian Massif	92	Abg44
Stara Planina Mts.	111	Aro14, Aro15
Stara Planina Mts.	112	gd28rs
Stara Planina Mts.	113	Abg04
Stara Planina Mts.	114	Abg43
Stara Planina Mts.	115	gd29rs
Stara Planina Mts.	116	Abg41
Stara Planina Mts.	117	Abg33
Stara Planina Mts.	118	Abg10, Abg11
Stara Planina Mts.	119	Abg23
Stara Planina Mts.	123	Abg30
Stara Planina Mts.	124	Abg05, Abg06
Stara Planina Mts.	125	Abg21
Stara Planina Mts.	126	Abg12
