# Supplementary material for: Contrasting evolutionary histories of the legless lizards slow worms (Anguis) shaped by the topography of the Balkan Peninsula
Source: BMC Evol Biol. 2016 May 10;16:99. doi: 10.1186/s12862-016-0669-1 (PMC4863322; doi:10.1186/s12862-016-0669-1)

Additional file 3: Figure S1. A map of demarcated topographic units as defined for regression analyses of nucleotide diversity (π) and terrain ruggedness index (TRI).

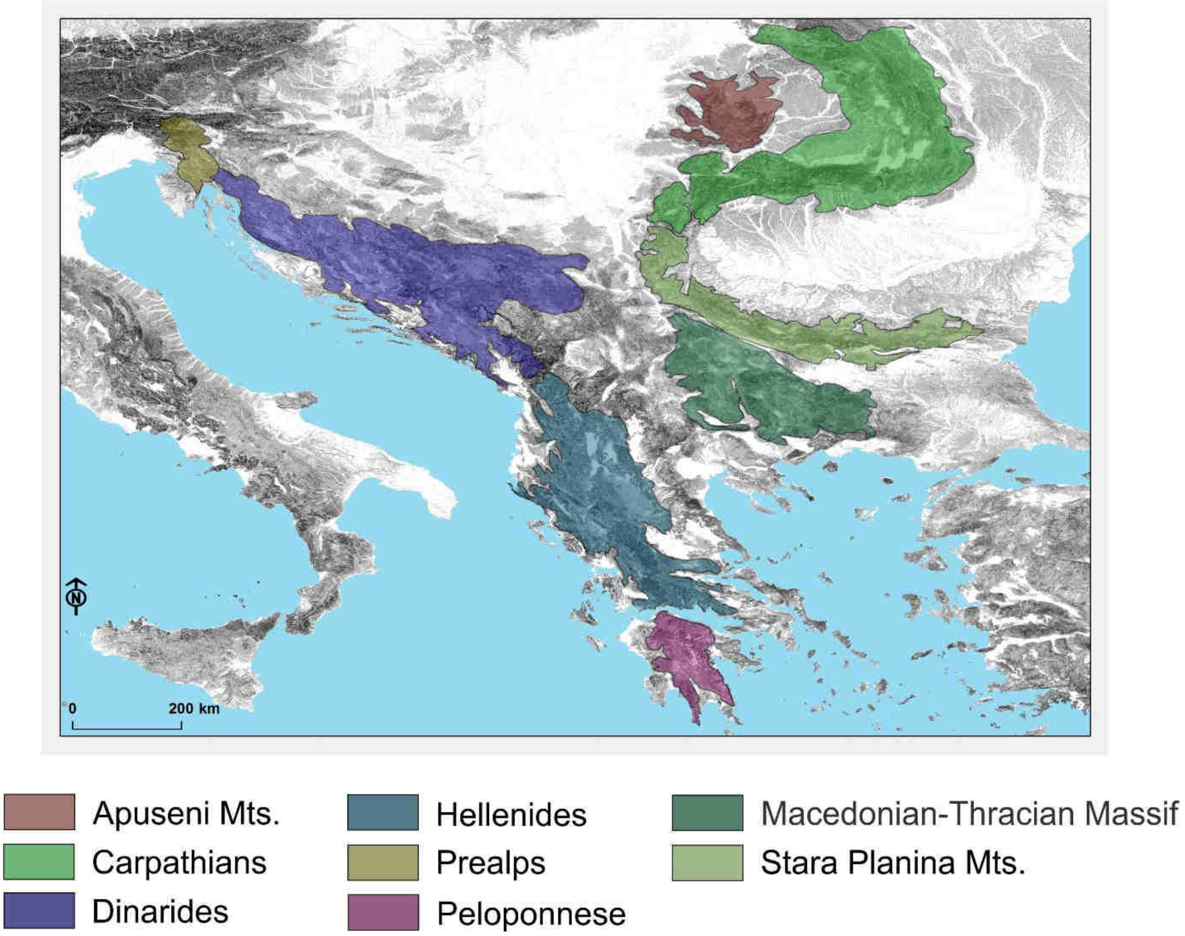

Supplement: Additional file 3: Figure S1. — A map of demarcated topographic units as defined for regression analyses of nucleotide diversity (π) and terrain ruggedness index (TRI). (PDF 131 kb) [file 12862_2016_669_MOESM3_ESM.pdf]
