# Supplementary material for: Contrasting evolutionary histories of the legless lizards slow worms (Anguis) shaped by the topography of the Balkan Peninsula
Source: BMC Evol Biol. 2016 May 10;16:99. doi: 10.1186/s12862-016-0669-1 (PMC4863322; doi:10.1186/s12862-016-0669-1)
Supplement: Additional file 4: Table S3. — Values of nucleotide diversity (π), area size of particular topographic units (in km2), and terrain ruggedness index (TRI). The third quartile (TRI Q3), and median (TRI Q3 median) and modus (TRI Q3 modus) of data above TRI Q3 were used in regression analyses. (PDF 21 kb) [file 12862_2016_669_MOESM4_ESM.pdf]

Additional file 4: Table S3. Values of nucleotide diversity (π), area size of particular topographic units (in km^2) and terrain ruggedness index (TRI). The third quartile (TRI Q3), and median (TRI Q3 median) and modus (TRI Q3 modus) of data about TRI Q3 were used in regression analyses.

**Mountain units**	**nucleotide diversity (π)**	**Area (km^2)**	**TRI Q3**	**TRI Q3 median**	**TRI Q3 modus**
Apuseni Mts.	0.073	1102.9175	67.3238	83.5464	67.8307
Carpathians	0.489	6537.1879	70.4486	88.0625	81.0432
Dinarides	0.233	5856.8021	67.5870	88.0000	67.6757
Hellenides	1.202	4491.3329	85.4225	108.1457	93.1289
Prealps	0.594	540.6640	75.9803	105.7071	77.9743
Peloponnese	0.907	1073.7173	86.0000	106.2003	96.8504
Macedonian-Thracian Massif	0.123	3218.7286	75.3260	91.6570	75.4785
Stara Planina Mts.	0.098	2704.3606	67.3424	84.1368	68.3228
Apuseni Mts. + Carpathians	0.454	7640.1053	69.9643	87.3184	74.6994
