# Supplementary material for: Contrasting evolutionary histories of the legless lizards slow worms (Anguis) shaped by the topography of the Balkan Peninsula
Source: BMC Evol Biol. 2016 May 10;16:99. doi: 10.1186/s12862-016-0669-1 (PMC4863322; doi:10.1186/s12862-016-0669-1)
Supplement: Additional file 5: Table S4. — Average uncorrected p-distances calculated among the main evolutionary lineages within each of the four Anguis species distributed in the Balkans. The highest values are in bold. (PDF 13 kb) [file 12862_2016_669_MOESM5_ESM.pdf]

Additional file 5: Table S4. Average uncorrected *p*-distances calculated among the main evolutionary lineages within each of the four *Anguis* species distributed in the Balkans. The highest values are in bold.

A. cephallonica

	Mani lineage	Widespread lineage
Mani lineage	-	
Widespread lineage	2.4	-

A. fragilis

	Carniolan	Alpine-Pannonian	North Adriatic	Illyrian-Central European	South Balkan
Carniolan	-				
Alpine-Pannonian	0.6	-			
North Adriatic	0.9	0.9	-		
Illyrian-Central European	0.9	1.0	1.0	-	
South Balkan	1.0	1.0	**1.1**	0.3	-

A. colchica

	COLCHICA clade	ORIENTALIS clade	PONTIC clade	INCERTA clade
COLCHICA clade	-			
ORIENTALIS clade	3.6	-		
PONTIC clade	**4.7**	3.8	-	
INCERTA clade	3.6	3.1	4.3	-

	Stara-Planina lineage	Banatian lineage	Carpathian lineage
Stara-Planina lineage	-		
Banatian lineage	0.8	-	
Carpathian lineage	**1.0**	0.9	-

A. graeca

	graeca XII	*graeca* XI	*graeca* X	*graeca* IX	*graeca* VIII	*graeca* VII	*graeca* VI	*graeca* V	*graeca* IV	*graeca* III	*graeca* II	*graeca* I	KJ634800	KJ634801
graeca XII	-													
graeca XI	1.2	-												
graeca X	1.2	0.8	-											
graeca IX	0.8	1.2	1.2	-										
graeca VIII	1.0	1.4	1.4	1.0	-									
graeca VII	1.8	1.9	1.6	1.8	1.9	-								
graeca VI	1.5	2.2	1.9	1.8	1.9	1.6	-							
graeca V	1.3	1.8	1.5	1.4	1.4	1.2	0.9	-						
graeca IV	1.4	1.8	1.4	1.4	1.3	1.6	1.6	1.1	-					
graeca III	1.2	1.6	1.6	1.2	1.1	1.6	1.7	1.2	0.8	-				
graeca II	1.6	2.0	1.7	1.6	1.4	1.7	1.7	1.2	0.8	0.9	-			
graeca I	1.4	1.8	1.6	1.4	1.3	1.5	1.6	0.9	0.7	0.7	0.8	-		
KJ634800	2.6	3.3	3.0	2.9	2.5	3.0	2.7	2.3	1.8	2.2	2.3	1.7	-	
KJ634801	3.1	**3.6**	3.3	3.1	3.0	3.1	3.3	2.6	2.3	2.5	2.5	1.9	2.5	-
